# Comparative Analysis of Mitogenomes in Leafhopper Tribe Deltocephalini (Hemiptera: Cicadellidae: Deltocephalinae): Structural Conservatism and Phylogeny

**DOI:** 10.1002/ece3.70738

**Published:** 2024-12-18

**Authors:** Bingqing Xie, Xinyi Zhang, Yongxia Zhang, Christopher H. Dietrich, Yani Duan

**Affiliations:** ^1^ Anhui Province Key Laboratory of Integrated Pest Management on Crops, Key Laboratory of Biology and Sustainable Management of Plant Diseases and Pests of Anhui Higher Education Institutes, School of Plant Protection Anhui Agricultural University Hefei Anhui China; ^2^ Illinois Natural History Survey, Prairie Research Institute University of Illinois Champaign Illinois USA

**Keywords:** Cicadellidae, Deltocephalini, Hemiptera, molecular biology, phylogeny

## Abstract

Previous studies on the gene order and composition of leafhopper mitochondrial genomes have revealed a high level of conservation in overall genome structure. Some members of Deltocephalinae, the largest subfamily, exhibit tRNA gene rearrangements; however, few mitogenomes have been sequenced in this group and the degree of structural variation within tribes remains unclear. In this study, we sequenced the complete mitogenomes of 14 species belonging to four genera of tribe Deltocephalini from China and compared them with the two previously reported mitogenomes for this tribe. The studied mitogenomes showed a high degree of similarity to most other leafhopper mitogenomes in overall structure, mostly varying in the total length (14,961–15,416 bp) and number of non‐coding A + T‐rich regions. Gene size, order, arrangement, base composition, codon usage, and secondary structure of tRNAs in the newly sequenced mitogenomes were highly conserved in Deltocephalini, and variations in start/stop codon usage and tRNA secondary structure mostly matched those of other leafhoppers. Phylogenetic analysis of different combinations of protein‐coding and ribosomal genes using maximum likelihood and Bayesian inference under different models using either amino acid or nucleotide sequences were generally consistent and agreed with the previous nuclear and partial mitochondrial gene sequence data, indicating that complete mitochondrial genomes are phylogenetically informative at different levels of divergence within Deltocephalini and among different leafhoppers species. In addition to Deltocephalini, Deltocephalinae included members of Athysanini and Opsiini formed monophyletic groups. Maximum likelihood and Bayesian inference analyses consistently grouped *Graminella nigrinota* with Paralimnini, rendering Deltocephalini polyphyletic. The topology consistently divided Deltocephalini into two major branches, with *Alobaldia tobae* and *Polyamia penistenuis* forming a well‐supported sister group to the remaining species of the tribe.

## Graphical abstract

In this study, we sequenced the complete mitogenomes of 14 species belonging to four genera of tribe Deltocephalini from China and compared them with the two previously reported mitogenomes for this tribe. Phylogenetic analysis of different combinations of protein‐coding and ribosomal genes using maximum likelihood and Bayesian inference under different models using either amino acid or nucleotide sequences were generally consistent and agreed with the previous nuclear and partial mitochondrial gene sequence data, indicating that complete mitochondrial genomes are phylogenetically informative at the different levels of divergence within Deltocephalini and among different leafhoppers species.

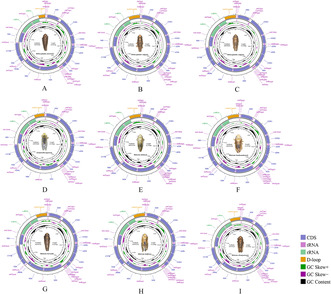

## 1 Introduction

Mitogenome sequences are widely used in studies on insect molecular evolution, phylogenetic relationships, and species identification because of their maternal inheritance, relatively high evolutionary rates, low levels of recombination, high genome copy numbers, and conserved gene components (Abascal et al. 2006; Cameron 2014; Wilson et al. 1985).

In leafhoppers (Cicadellidae), mitogenome sequences have been incorporated into several recent phylogenetic studies and used to resolve relationships among lineages at various taxonomic levels (Wang et al. 2020; Du et al. 2021; Lin, Huang, and Zhang 2021; Yu and Zhang 2021; Shah et al. 2024). Although leafhopper mitogenomes exhibit highly conserved overall structure, there may be rearrangements of tRNA genes that are phylogenetically informative. Therefore, complete mitogenome sequences of leafhoppers may enhance our understanding of the evolution of this highly diverse herbivore group.

Grass‐specialist leafhopper tribe Deltocephalini presently comprises 601 described species in 76 genera distributed worldwide (Dmitriev et al. 2022); however, some large clades are restricted to specific biogeographic regions (Cao et al. 2022). Recent phylogenetic studies strongly support the monophyly of this tribe; however, the relationships among genera within this tribe and among regional fauna remain inadequately resolved. In China, Deltocephalini comprises 60 described species in 14 genera: *Maiestas* Distant (11 species), *Deltocephalus* Burmeister (7 spp.), *Paramesodes* Ishihara (6 spp.), *Polyamia* DeLong (2 spp.), *Yuanamia* Zhang (2 spp.), *Alobaldia* Emeljanov (1 sp.), *Ctenurellina* McKamey (1 sp.), *Endria* Oman (1 sp.), *Graminella* DeLong (1 sp.), *Matsumuratettix* Metcalf (1 sp.), *Peitouellus* Vilbaste (1 sp.), *Pseudomaiestas* Xu, Shah & Duan (1 sp.), *Recilia* Edwards (1 sp.), and *Wyushinamia* Zhang & Duan (1 sp.). Some species of Deltocephalini are important agricultural pests, causing damage through feeding and transmitting the plant pathogens. For example, *Maiestas dorsalis* transmits the rice stripe mosaic virus, rice gall dwarf virus, and rice orange leaf phytoplasma (Chen et al. 2016, 2023; Zhao et al. 2019), *Graminella nigrifrons* transmits the maize chlorotic dwarf virus (Cassone et al. 2014; Catto et al. 2022), and *Endria inimica* transmits the wheat striate mosaic virus (Sinha and Chiykowski 1967).

Previous phylogenetic studies have included various Deltocephalini species. Fang et al. (1993) only included New World taxa and partial mitochondrial *16S rDNA* sequences. Fang, Blocker, and Black (1995) subsequently incorporated additional sequence data from *Cyt b*, *tRNA Ser*, and *ND1* mitochondrial genes combined with morphology, yielding a well‐resolved phylogeny but with low branch support and again focusing only on New World taxa. Kamitani (1999) conducted a phylogenetic analysis of Japanese Deltocephalini and Paralimnini based on data from 64 morphological characters and moved some genera of Deltocephalini to Paralimnini. Zahniser and Dietrich (2010) conducted a phylogenetic analysis of Deltocephalinae using combined sequence data from *28S rDNA* and *H3* with morphological characteristics, yielding a well‐resolved tree, but with many branches poorly supported. The analysis by Dietrich et al. (2017), based on anchored hybrid enrichment data, included only one species of Deltocephalini. The mitogenome‐based analyses of Song, Cai, and Li (2017); Song, Zhang, and Zhao (2019) focused on the relationships among major Cicadomorpha lineages using mitogenome sequences but included only 1–6 species of the tribe. Zhang, Zhang, and Duan (2019) analyzed the phylogeny of *Deltocephalus* using the barcode region of the mitochondrial *COX1* gene, showing a barcode gap among species recognized based on morphology. Some analyses of Cicadomorpha, Auchenorrhyncha, and Membracoidea used larger amounts of sequence data, but included few representatives of Deltocephalini. Skinner et al. (2020) conducted a phylogenomic analysis of Auchenorrhyncha based on transcriptome sequences but also included only one species of Deltocephalini. The comprehensive phylogenomic analysis of Cao et al. (2022), based on anchored hybrid enrichment data from 730 terminal taxa and > 160,000 nucleotide positions, supported the monophyly of Deltocephalini (except *Loeia* Duan and *Yuanamia* Zhang & Duan) and grouped New and Old World taxa into distinct lineages. A transcriptome‐based analysis of Membracoidea by Hu et al. (2023) included only two species of the tribe. Wu et al. (2023) recently reconstructed the interspecific phylogenetic relationships of Deltocephalini using a combination of mitochondrial (*COX1*) and nuclear (*28S rDNA*, *Wingless* and *H3*) genes for 32 species in eight genera of the tribe and revealed that some recognized genera, previously defined based on morphology (*Deltocephalus*, *Maiestas*, and *Polyamia*), are not monophyletic.

Previously, only two complete mitogenomes of Deltocephalini were available, and complete mitogenome sequences have not been used to elucidate the relationships within Deltocephalini (Du et al. 2017; Song, Cai, and Li 2017). Therefore, in this study, we sequenced 15 additional Deltocephalini mitogenomes, conducted comparative mitogenomic analyses and used the available mitogenome sequence data to reconstruct the phylogenetic relationships between Deltocephalini and other tribes of subfamily Deltocephalinae.

## 2 Materials and Methods

### 2.1 Taxa Sampling and Species Identification

Leafhopper samples were collected between 2016 and 2021 in Anhui, Guangxi, Guizhou, Yunnan and Zhejiang Provinces, China (Table 1). All samples were collected from the field and did not require any permits. Fresh samples were preserved in 95% ethanol and stored at −40°C. prior to DNA extraction, the samples were identified to species based on external morphology and male genitalia using available taxonomic literature. Specimens were studied using Nikon SMZ1500 and Eclipse 50I microscopes (Nikon Corporation, Tokyo, Japan). Included specimens are listed in Table 1 and are deposited at Anhui Agricultural University, Hefei, China (AAU).

**TABLE 1:** Specimens and previously published data of the Deltocephalini species used in this study (all from China).

Organism	Locality	Code	GenBank accession	Reference
*Alobaldia tobae*			KY039116	Song, Cai, and Li (2017)
*Deltocephalus uncinatus*	Guizhou	GZy05	OP978288	This study
*Deltocephalus vulgaris*	Guangxi	GXd02	OP978289	This study
*Deltocephalus vulgaris*	Yunnan	YNf31	OP978291	This study
*Graminella nigrinota*	Yunnan	YNi07	OP978290	This study
*Maiestas distincta*	Anhui	AHF01	OP972719	This study
*Maiestas dorsalis*			KX786285	Du et al. (2017)
*Maiestas heuksandoensis*	Yunnan	YNo17	OP972720	This study
*Maiestas horvathi*	Zhejiang	ZJk08	OP972721	This study
*Maiestas latifrons*	Guizhou	GZy08	OP972722	This study
*Maiestas obongsanensis*	Guizhou	GZy18	OP972723	This study
*Maiestas oryzae*	Anhui	AH0118	OP972724	This study
*Maiestas remigia*	Yunnan	YNm31	OP972725	This study
*Maiestas subviridis*	Yunnan	YNn31	OP972726	This study
*Maiestas tareni*	Yunnan	YNf03	OP972727	This study
*Maiestas yangae*	Anhui	AH0007	OP972728	This study
*Polyamia penistenuis*	Anhui	AH0019	OP972729	This study

### 2.2 DNA Extraction and Sequencing

The DNA extraction and high‐throughput sequencing of the samples were performed by Shanghai Personalbio Company. The mitogenomes were sequenced using high‐throughput sequencing on an Illumina NovaSeq platform (United States Illumina Company, San Diego, CA, USA) for each species, generating approximately 3 GB of data per sample with a read length of 150 bp. The raw data were subjected to quality control and filtering (removing adapter contamination, trimming reads shorter than 50 bp, removing reads with an average quality score lower than 20, and removing reads with more than 3 Ns), resulting in high‐quality data for subsequent analysis.

### 2.3 Mitogenome Sequence Assembly, Annotation and Analysis

The mitogenome sequences were assembled and annotated using GENEIOUS (version 8.1.3) (Kearse et al. 2012). The assembled sequences were compared with published mitochondrial genome sequences of other Cicadellidae using the built‐in MAFFT plugin in GENEIOUS. The sequences of 37 individual genes, including the 13 PCGs, 22 tRNAs, and 2 rRNAs, were extracted from each mitogenome. The lengths and positions of the 13 protein‐coding genes were confirmed by comparing them to multiple reference sequences, and the start and stop codons were identified within the open reading frames to annotate the protein‐coding genes. The annotation of RNA genes and the prediction of tRNA secondary structure were performed using the Mitos online tool (http://mitos.bioinf.uni‐leipzig.de/index.py, accessed on April 10, 2024) (Bernt et al. 2013). The assembled mitochondrial genome sequence was uploaded and analyzed using the invertebrate mitochondrial codon table to determine start and stop positions of RNA genes. The prediction of tRNA secondary structures was also conducted on the same website. After annotation, the mitochondrial genome structure was visualized using the Proksee online website (https://proksee.ca/).

The annotated sequence file was imported into PhyloSuite (version 1.2.2) to extract annotation information. The relative synonymous codon usage (RSCU) of protein‐coding genes and nucleotide composition and were analyzed using PhyloSuite (Zhang et al. 2020). Base usage bias was calculated using the formulas: AT skew = (A − T)/(A + T) and GC skew = (G − C)/(G + C) (Perna and Kocher 1995). The genetic distances of the protein‐coding gene sequences among samples were analyzed using MEGA (version 7.0) with the Kimura 2‐parameter model. Non‐synonymous and synonymous substitution rates and sliding window analyses of protein‐coding gene sequences were calculated using DnaSP (version 6.0) (Rozas et al. 2017) using default settings, with a 200 bp sliding window and 20 bp overlap.

### 2.4 Phylogenetic Analysis

After integrating mitochondrial genome data and removing stop codons, protein‐coding genes and ribosomal RNA genes were aligned using MAFFT (version 7.520) (Katoh and Standley 2013), applying the G‐strategy to protein‐coding genes and the Q‐strategy to ribosomal RNA genes. Gblocks (version 0.91b) was used to remove gaps and ambiguous positions under default settings (Talavera and Castresana 2007). Five datasets were obtained by concatenating sequence files using PhyloSuite (version 1.2.2). Substitution Saturation were conducted using DAMBE (version 5.3.48) (Xia 2013), importing the concatenated sequence files and choosing the Xia method for testing. The heterogeneity of sequence divergence within the dataset was analyzed using AliGROOVE (version 1.0.8) (Kück et al. 2014), with the default sliding window size. Indels in the nucleotide dataset were treated as ambiguity and a BLOSUM62 matrix was used as the default amino acid substitution matrix.

PartitionFinder 2.1.1 (Lanfear et al. 2017) was used to determine the optimal models for constructing phylogenetic trees, selecting the best substitution models for the first, second, and third codon positions of protein‐coding genes using the “Al” option for both Maximum Likelihood (ML) and Bayesian Inference (BI) methods (Table S1). The Maximum Likelihood (ML) tree was constructed using IQ‐TREE (version 2.2.0) (Minh et al. 2020) by importing the partitioned model, selecting the ultrafast bootstrap (UFB) algorithm, and evaluating the support of each node based on 1000 replicates. The Bayesian Inference (BI) tree was constructed using MrBayes (version 3.2.7) (Ronquist et al. 2012) on the CIPRES Science Gateway (www.phylo.org) by importing the partitioned model, running 4 MCMC chains for 40 million generations, with a sampling frequency of 1000 generations. The BI tree construction was stopped after convergence, as determined by an average standard deviation of split frequencies below 0.01, and the first 25% of trees were discarded as burn‐in. A consensus tree was then calculated to evaluate the posterior probability of each node.

## 3 Results and Discussion

### 3.1 Mitogenome Analysis

In this study, 15 mitogenomes representing 14 species were sequenced for the leafhoppers of tribe Deltocephalini from China. Comparative mitochondrial genomic analysis was performed for both the newly sequenced and two previously published mitogenomes, resulting in 16 Deltocephalini species subjected to further phylogenetic analysis (Table 1).

### 3.2 Mitogenome Organization and Gene Content

**FIGURE 1:**
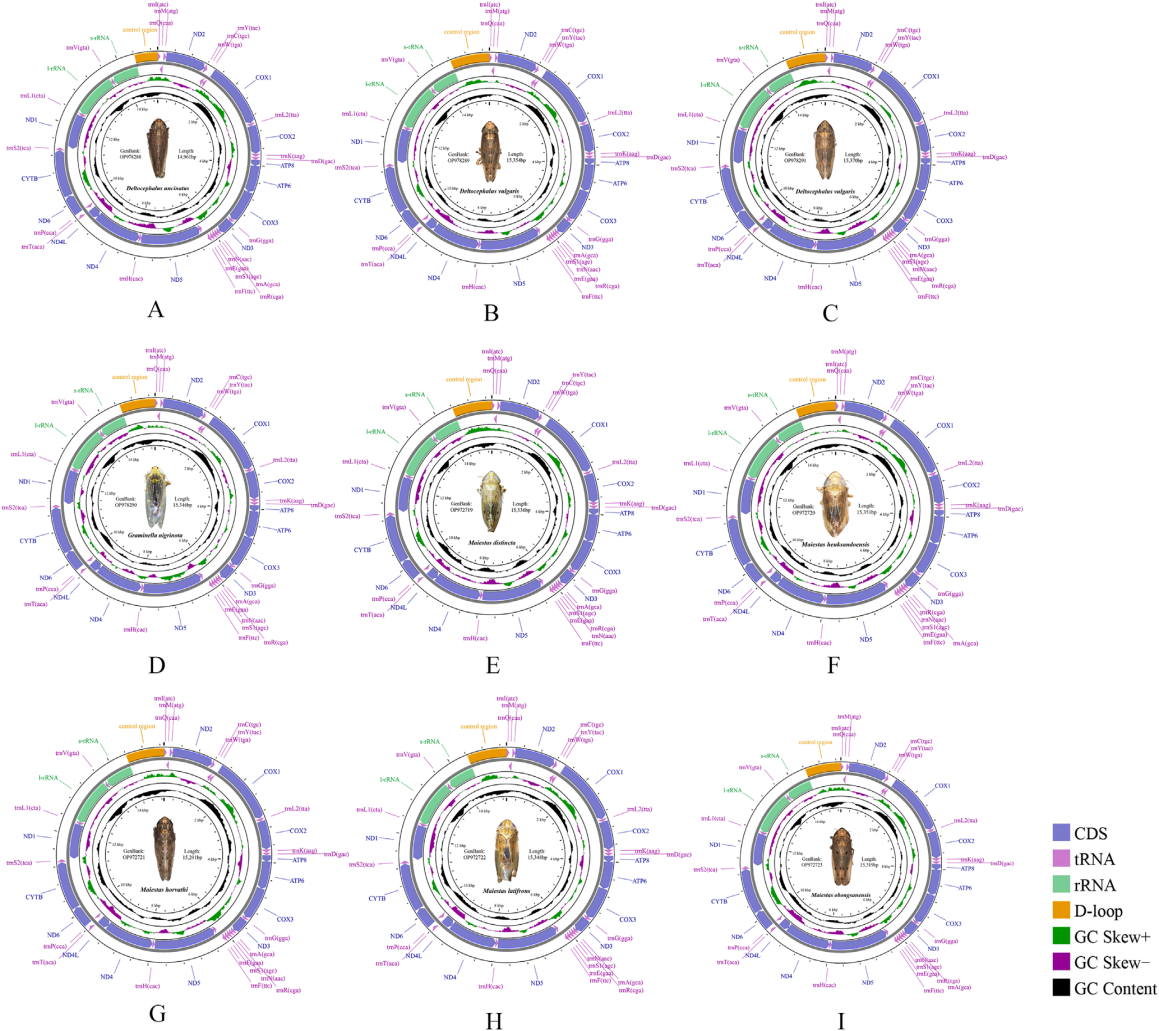
Circular maps of the complete mitochondrial genomes of various Deltocephalini species. (A) *Deltocephalus uncinatus*. (B) *Deltocephalus vulgaris* (OP978289). (C) *Deltocephalus vulgaris* (OP978291). (D) *Graminella nigrinota*. (E) *Maiestas distincta*. (F) *Maiestas heuksandoensis*. (G) *Maiestas horvathi*. (H) *Maiestas latifrons*. (I) *Maiestas obongsanensis*.

**FIGURE 2:**
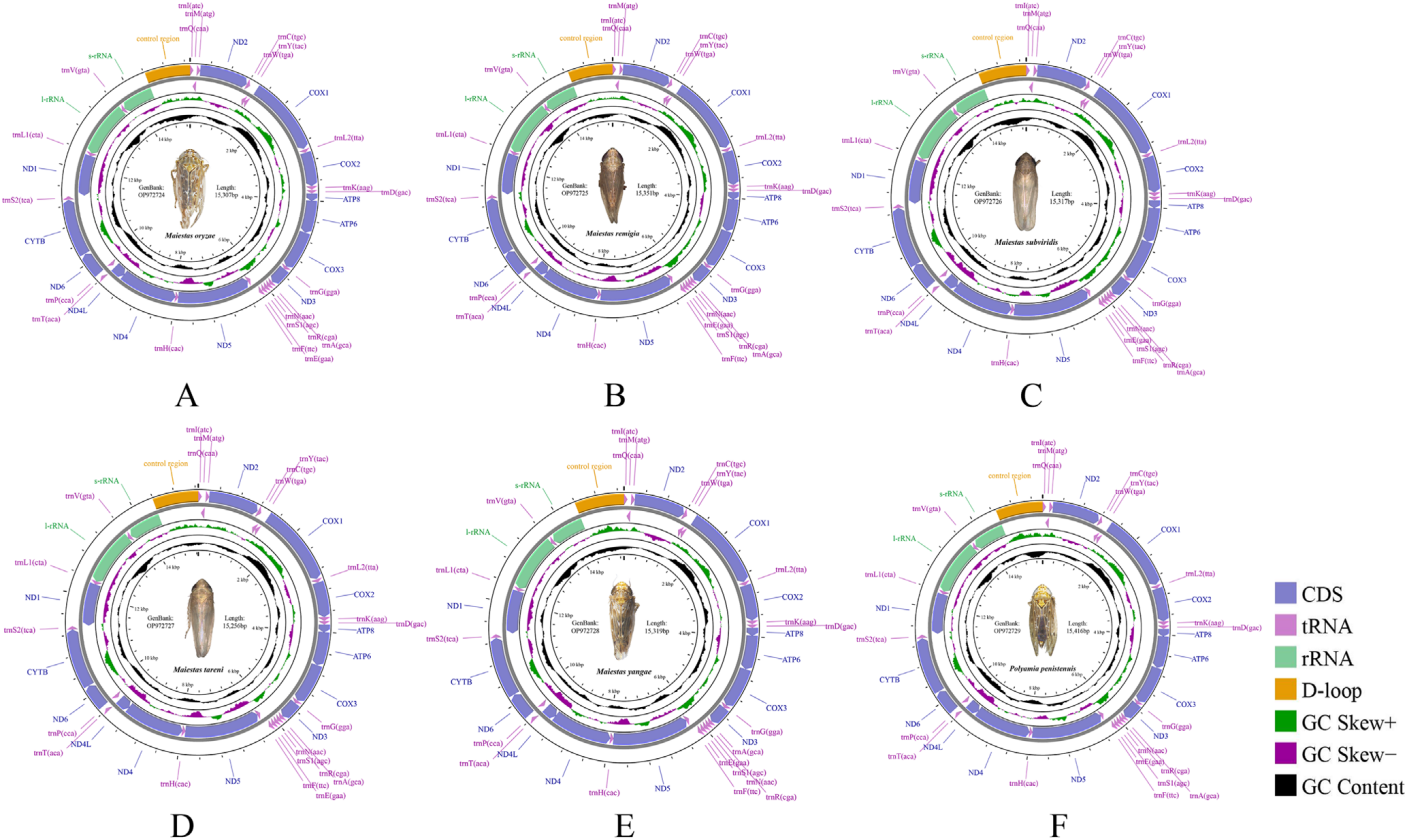
Circular maps of the complete mitochondrial genomes of different Deltocephalini species. (A) *Maiestas oryzae*. (B) *Maiestas remigia*; (C) *Maiestas* *subviridis*. (D) *Maiestas tareni*. (E) *Maiestas yangae*. (F) *Polyamia penistenuis*.

Gene composition, order, and structure of all 17 (16 species) sequenced Deltocephalini mitochondrial genomes closely resembled those of most other sequenced leafhoppers (Figures 1 and 2). *A. tobae* control region (CR) was incomplete due to the presence of a complex structure or high AT content, resulting in the presence of a 100‐bp long gap. The total length of the remaining 16 complete mitochondrial genome sequences ranged from 14,961 to 15,416 bp, with the shortest for *Deltocephalus uncinatus* (14,961 bp) and the longest genome observed for *P. penistenuis* (15,416 bp) (Table 2). The 13 protein‐coding genes (PCGs) were 10,937–10,996 bp in length. The 22 tRNA genes were 1447–1477 bp in length. The *rrnL* gene was 1209–1237 bp in length. The *rrnS* gene was 739–758 bp in length. CR was the most variable, ranging from 546 to 946 bp in length, and was the main factor affecting the variation in mitochondrial genome size. We did not detect any gene rearrangements in the Deltocephalini. Mitochondrial gene rearrangements are rare among leafhoppers but have been reported in some Deltocephalinae tribes (Du, Dietrich, and Dai 2019).

**TABLE 2:** Lengths and base compositions of various Deltocephalini mitochondrial genomes analyzed in this study.

Organism	Length (bp)	AT%	AT Skew	GC Skew
*Alobaldia tobae*	16,026	77.8	0.123	−0.162
*Deltocephalus uncinatus*	14,961	77.7	0.125	−0.188
*Deltocephalus vulgaris* OP978289	15,354	76.8	0.138	−0.181
*Deltocephalus vulgaris* OP978291	15,370	76.9	0.134	−0.169
*Graminella nigrinota*	15,346	74.6	0.129	−0.126
*Maiestas distincta*	15,336	77.0	0.143	−0.170
*Maiestas dorsalis*	15,352	78.7	0.128	−0.160
*Maiestas heuksandoensis*	15,351	78.2	0.125	−0.180
*Maiestas horvathi*	15,291	76.3	0.119	−0.181
*Maiestas latifrons*	15,348	78.0	0.126	−0.182
*Maiestas obongsanensis*	15,319	77.6	0.137	−0.193
*Maiestas oryzae*	15,307	77.9	0.130	−0.176
*Maiestas remigia*	15,351	77.7	0.140	−0.202
*Maiestas subviridis*	15,317	77.9	0.130	−0.167
*Maiestas tareni*	15,256	77.9	0.127	−0.167
*Maiestas yangae*	15,319	76.1	0.122	−0.176
*Polyamia penistenuis*	15,416	75.6	0.135	−0.197

### 3.3 Nucleotide Composition

**FIGURE 3:**
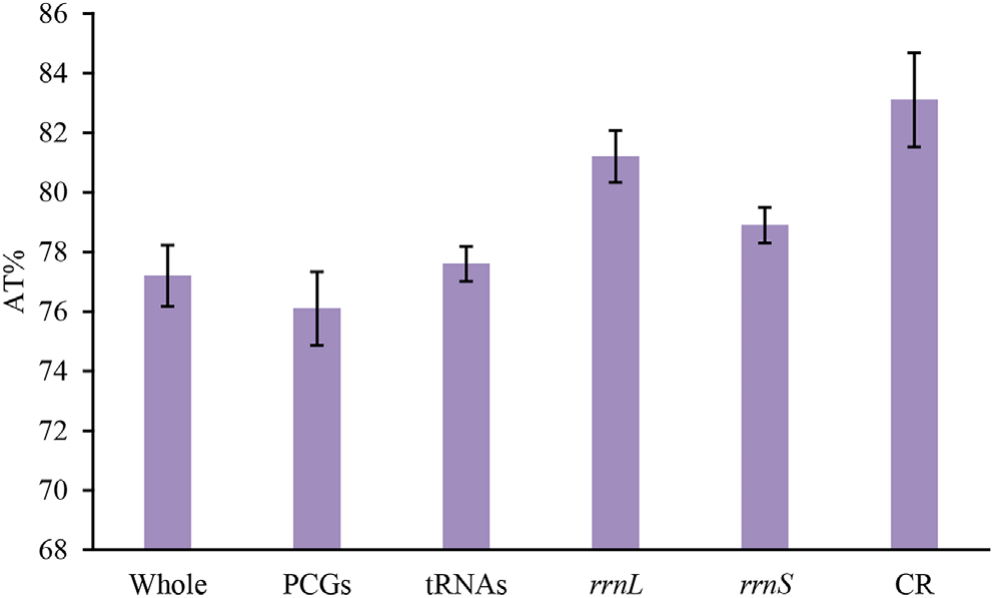
AT% in different regions of the Deltocephalini mitochondrial genomes.

AT content of the 17 Deltocephalini mitochondrial genomes was 74.6%–78.7%, with each component exhibiting a relatively high AT content. PCGs exhibited the lowest AT content, followed by tRNAs, *rrnS*, and *rrnL*. The highest AT content was found in CR, with a mean value exceeding 83%. This indicated a clear AT bias in the complete mitogenome of the Deltocephalini species (Table 2; Figure 3).

### 3.4 PCGs And Codon Usage

**FIGURE 4:**
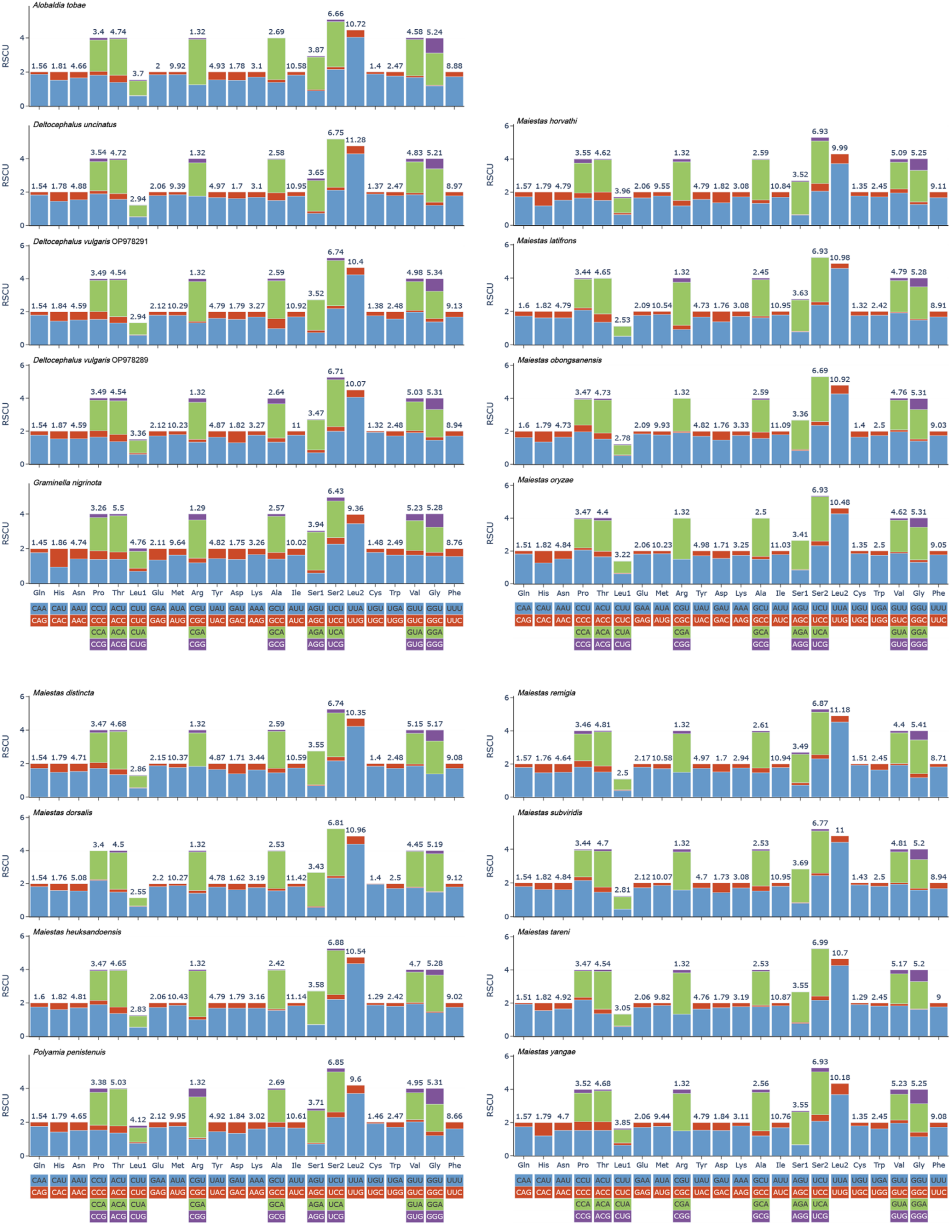
Relative synonymous codon usage (RSCU) of the protein‐coding genes (PCGs) of various Deltocephalini species.

**FIGURE 5:**
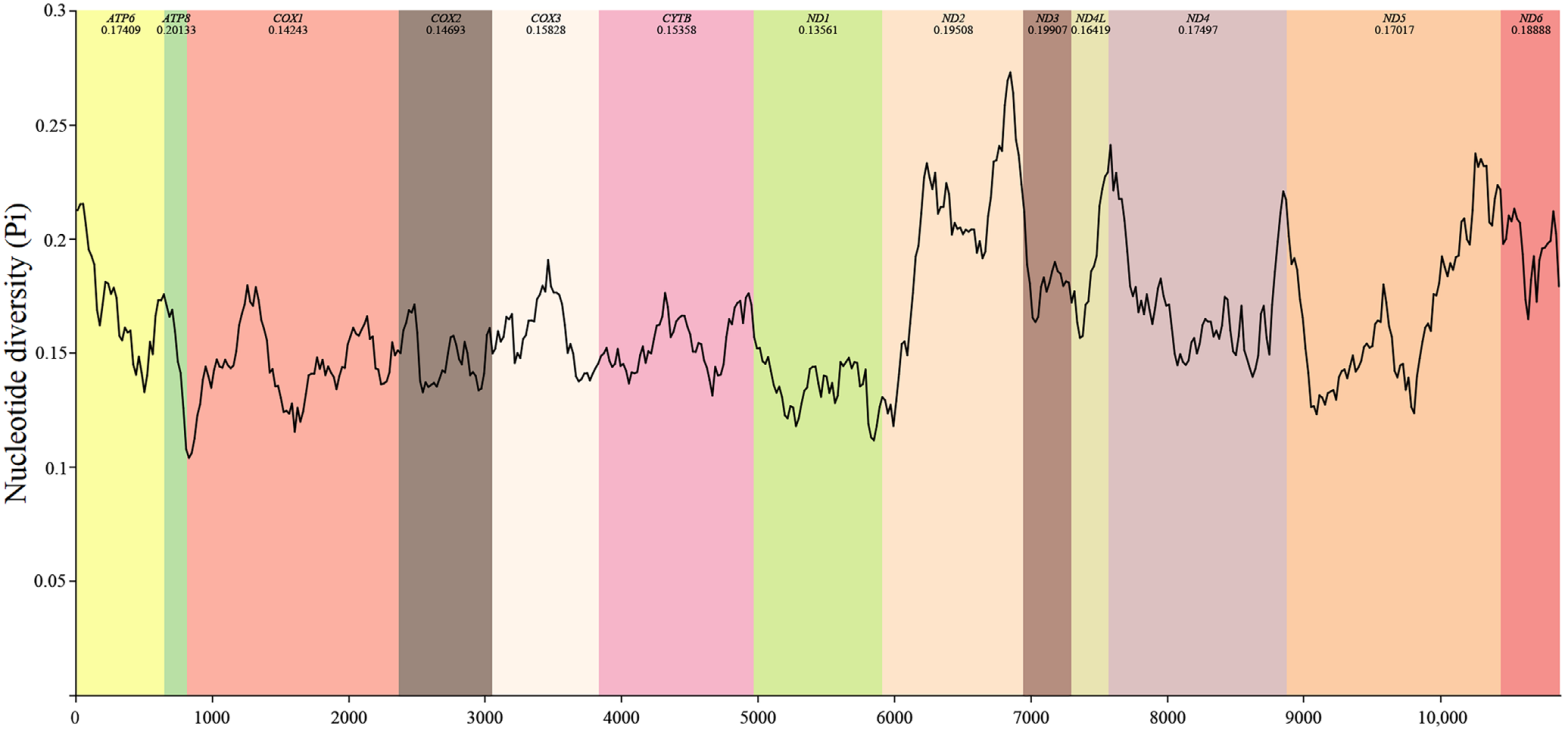
Sliding‐window analyses based on 13 aligned PCGs among various Deltocephalini species.

**FIGURE 6:**
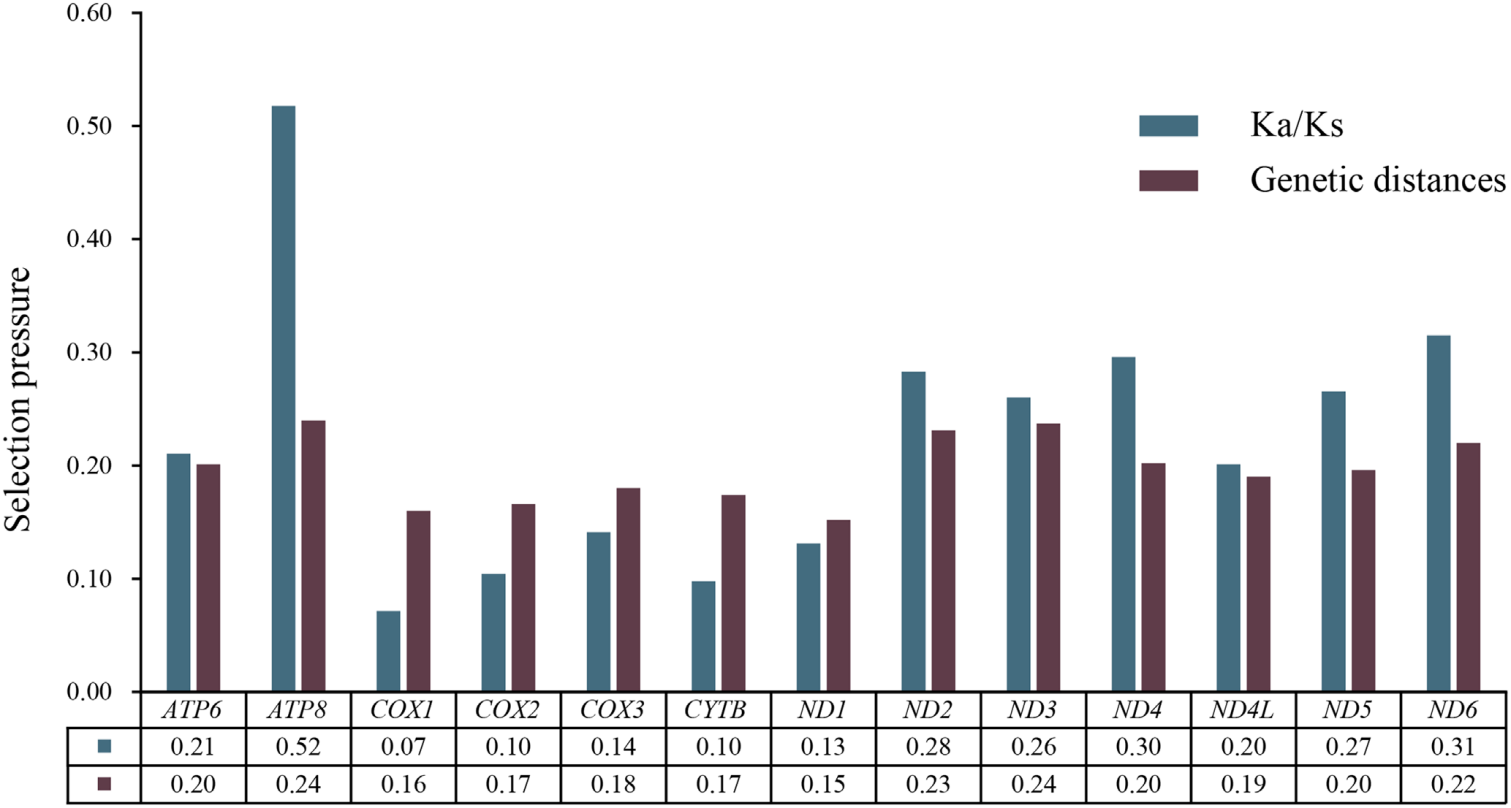
Genetic distances and ratios of non‐synonymous (Ka) to synonymous (Ks) substitution rates of 13 aligned PCGs among various Deltocephalini species. Average value for each PCG is shown below the gene name.

PCGs of Deltocephalini species exhibited five initiation codons: ATT (84 times), ATG (82 times), ATA (22 times), TTG (17 times), and ATC (16 times). Most stop codons were TAA or T but some were TAG or TA. Specifically, TAA was observed 168 times, T 30 times, TAG 22 times, and TA 1 times (Table 3). Statistical analysis of the relative synonymous codon usage showed that the most frequently observed amino acids in the Deltocephalini mitochondrial genome were Ile, Leu2, Met, and Phe (Figure 4). Based on the sliding window analysis of the 13 PCGs in the 16 Deltocephalini species, nucleotide diversity of *ATP8* (Pi = 0.201) was the highest. Notably, *CYTB* (Pi = 0.154), *COX2* (Pi = 0.147), *COX1* (Pi = 0.142), and *ND1* (Pi = 0.136) showed low nucleotide diversity (Figure 5). According to the genetic distance analysis of the 13 PCGs, *ATP8* (0.24) and *ND3* (0.24) exhibited the highest genetic distance and fastest evolution rate, whereas *ND1* (0.15) exhibited the lowest genetic distance and slowest evolution rate. The ratio of synonymous (Ks) to non‐synonymous (Ka) substitution rates (Ka/Ks, ω) among the 13 PCGs was 0.07–0.52 (0 < ω < 1). All ω values were < 1, indicating that the 13 PCGs were under purifying selection at the gene level. *COX1* exhibited the lowest ω value (0.07), whereas *ATP8* exhibited the highest ω value (0.52), with a significant difference (Figure 6).

**TABLE 3:** Use of start and stop codons of the mitochondrial protein‐coding genes (PCGs) of Deltocephalini species.

Species	Start codon/Stop codon (ATN/TAN)												
	*ATP6*	*ATP8*	*COX1*	*COX2*	*COX3*	*CYTB*	*ND1*	*ND2*	*ND3*	*ND4*	*ND4L*	*ND5*	*ND6*
*Alobaldia tobae*	G/A	T/A	A/A	A/T—	G/A	G/A	T/A	A/A	A/A	G/A	T/G	TTG/TA	T/A
*Deltocephalus uncinatus*	G/A	A/A	C/A	T/T—	G/A	G/A	T/A	C/A	A/A	G/A	T/A	TTG/G	T/A
*Deltocephalus vulgaris* OP978289	G/A	A/A	G/A	T/T—	G/A	G/A	T/A	T/A	T/A	G/A	T/A	TTG/G	T/G
*Deltocephalus vulgaris* OP978291	G/A	A/A	G/A	T/T—	G/A	G/A	T/T—	T/A	T/A	G/A	T/A	TTG/G	T/A
*Graminella nigrinota*	G/A	C/A	G/A	A/T—	A/A	G/A	T/A	C/A	A/G	G/G	T/A	TTG/A	G/A
*Maiestas distincta*	G/A	A/A	G/G	T/T—	G/A	G/A	T/A	T/A	A/A	G/A	T/G	TTG/G	T/A
*Maiestas dorsalis*	G/A	T/A	A/A	C/T—	G/A	G/A	T/T—	C/A	A/A	G/A	T/A	TTG/G	T/A
*Maiestas heuksandoensis*	G/A	T/A	G/A	T/T—	G/A	G/A	T/A	T/A	T/G	G/A	T/A	TTG/G	T/A
*Maiestas horvathi*	G/A	A/A	G/A	T/T—	G/A	G/A	T/T—	C/A	C/A	G/A	T/A	TTG/G	T/A
*Maiestas latifrons*	G/A	A/A	G/A	T/T—	G/A	G/A	T/T—	T/A	T/G	G/A	T/A	TTG/T—	T/A
*Maiestas obongsanensis*	G/A	T/A	G/A	T/T—	G/A	G/A	T/T—	C/A	A/A	G/A	T/A	TTG/G	T/A
*Maiestas oryzae*	G/A	T/A	G/A	T/T—	G/A	G/A	T/T—	C/A	A/A	G/T—	T/A	TTG/A	T/A
*Maiestas remigia*	G/A	T/A	G/A	T/T—	G/A	G/A	T/T—	T/A	A/A	G/A	T/G	TTG/G	T/A
*Maiestas subviridis*	G/A	A/A	G/A	C/T—	G/A	G/A	T/A	T/A	T/A	G/A	T/A	TTG/G	T/A
*Maiestas tareni*	G/A	A/A	G/A	T/T—	G/A	G/G	T/A	T/A	T/A	G/A	T/G	TTG/T—	T/A
*Maiestas yangae*	G/A	T/A	G/A	T/T—	G/A	G/A	T/T—	C/A	C/A	G/A	T/A	TTG/G	T/A
*Polyamia penistenuis*	G/A	C/A	G/A	C/T—	G/A	G/A	T/T—	T/A	T/A	G/A	T/A	TTG/T—	C/A

### 3.5 RNA Genes

**FIGURE 7:**
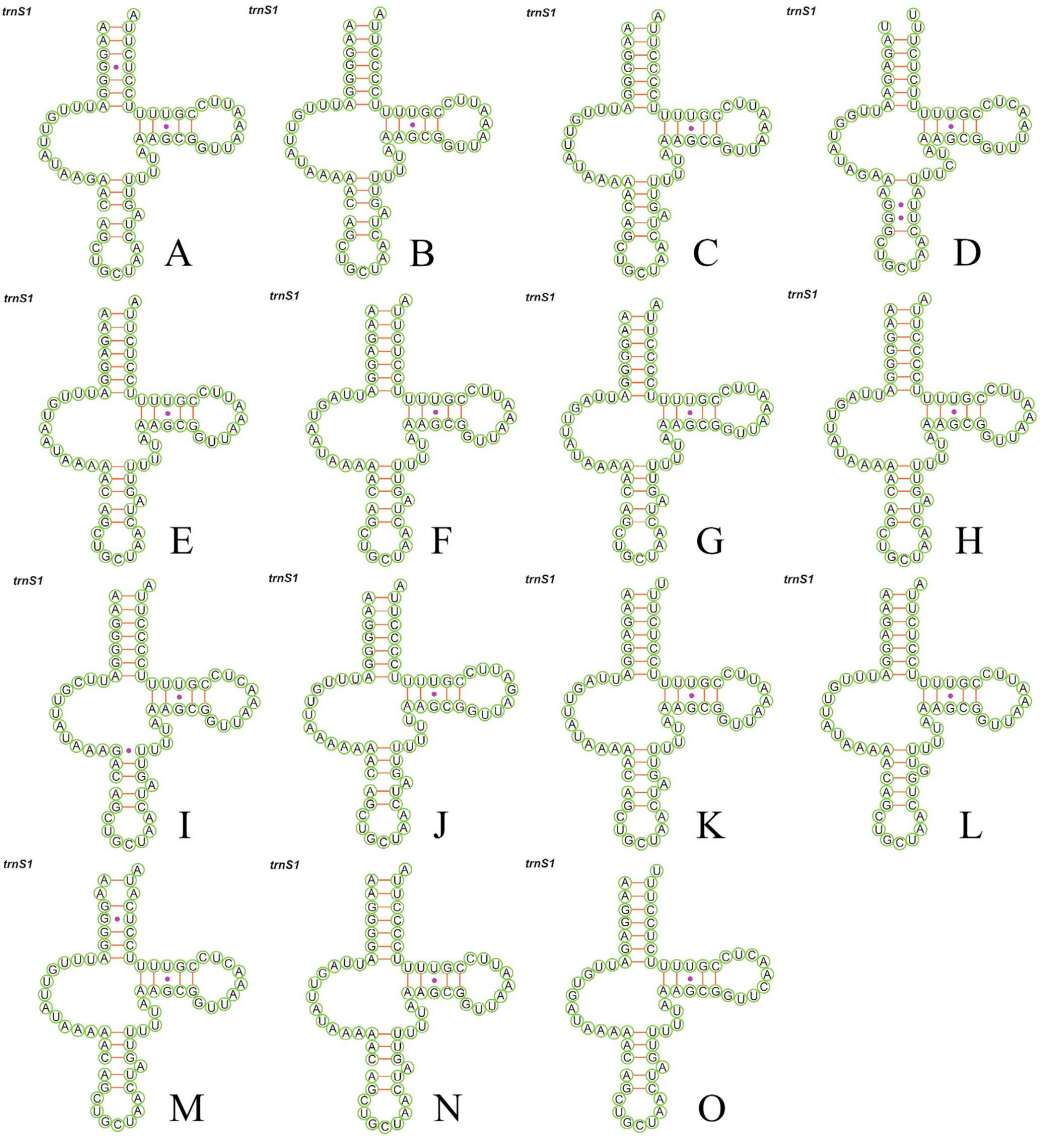
Putative secondary structures of *trnS1* in 15 Deltocephalini mitogenomes that do not possess the DHU‐arm. Dashes (−) indicate Watson−Crick base pairing, and dots (•) indicate G−U pairing. (A) *Deltocephalus uncinatus*. (B) *Deltocephalus vulgaris* (OP978289). (C) *Deltocephalus vulgaris* (OP978291). (D) *Graminella nigrinota*. (E) *Maiestas distincta*. (F) *Maiestas heuksandoensis*. (G) *Maiestas horvathi*. (H) *Maiestas latifrons*. (I) *Maiestas obongsanensis*. (J) *Maiestas oryzae*. (K) *Maiestas remigial*. (L) *Maiestas subviridis*. (M) *Maiestas tareni*. (N) *Maiestas yangae*. (O) *Polyamia penistenuis*.

The 22 tRNA genes of the Deltocephalini mitochondrial genome ranged in length from 57 to 73 bp. Dihydrouridine (DHU) arm of *trnS1* (AGN) was missing and formed a circular structure (Figure 7), whereas the remaining 21 tRNAs exhibited typical cloverleaf structures (Figures S1–, S15). In addition to the A‐U and G‐C mismatches, the predicted secondary structure of tRNA included several mismatches, including U–U, U‐C, A‐C, A‐A, A‐G, G‐U, G‐G. The *rrnL* length ranged from 1209 to 1237 bp, whereas the *rrnS* length ranged from 739 to 758 bp (Table 4).

**TABLE 4:** Comparison of transfer and mitochondrial RNAs in Deltocephalini mitochondrial genomes.

Species	tRNA base mismatch							*rrnL* (bp)	*rrnS* (bp)
	U–U	U‐C	A‐C	A‐A	A‐G	G‐U	G‐G		
*Alobaldia tobae*	3		1	3		26		1228	758
*Deltocephalus uncinatus*	2			2		22		1216	744
*Deltocephalus vulgaris* OP978289	4			2		22		1231	742
*Deltocephalus vulgaris* OP978291	3			2		25		1232	745
*Graminella nigrinota*	4		1	2		28	1	1225	744
*Maiestas distincta*			2	2	1	23		1219	747
*Maiestas dorsalis*	4	1		2		23		1217	745
*Maiestas heuksandoensis*	3		1	1		26		1222	748
*Maiestas horvathi*	3			2		26		1225	750
*Maiestas latifrons*	3			1		21		1224	747
*Maiestas obongsanensis*	2		1	1		23		1222	748
*Maiestas oryzae*	2		1	1		16		1221	739
*Maiestas remigia*	4			1		19		1236	753
*Maiestas subviridis*	2		1	2		26		1226	747
*Maiestas tareni*	3	1		2		27		1209	745
*Maiestas yangae*	3			2		27		1225	743
*Polyamia penistenuis*	6	1	1	3		23		1237	753

### 3.6 Gene Overlap and Non‐Coding Regions

Gene arrangement of the Deltocephalini mitochondrial genome was relatively compact, with overlapping regions ranging from 28 bp (*P. penistenuis*) to 58 bp (*A. tobae*). However, two stable gene overlaps between *ATP8* and *ATP6, ND4* and *ND4L*. The mitochondrial genome of Deltocephalini also contained intergenic spacer regions, the widest of which were found in *Maiestas oryzae* (16 sites, 101 bp). *A. tobae* exhibited the longest total length of intergenic spacer regions (157 bp), with the longest spacer being 68 bp long between *ND2* and *trnW*.

### 3.7 Phylogenetic Relationships

To estimate the relationships between leafhopper subfamilies, tribes in subfamily Deltocephalinae, and genera and species of Deltocephalini, we selected GenBank‐published representatives of Deltocephalinae and other Membracoidea subfamilies. Our dataset included 65 Membracoidea species (62 leafhoppers and three treehoppers) as the ingroup, and two Cicadoidea and two Cercopoidea species as the outgroups (Table 5).

**TABLE 5:** Summary of the mitochondrial genome data used for phylogenetic analysis.

Superfamily/Family/Subfamily	Tribe	Species	Accession number	Reference
Cercopoidea				
Cercopidae				
Callitettixinae	Callitettixini	*Callitettix braconoides*	JX844628	Liu et al. (2014)
Cercopinae	Cosmoscartini	*Cosmoscarta dorsimacula*	MK256929	Yan and Zu (2019)
Cicadoidea				
Cicadidae				
Cicadinae	Fidicinini	*Diceroprocta semicincta*	KM000131	GenBank
Cicadettinae	Lamotialnini	*Magicicada tredecula*	MH937695	Du et al. (2019)
Membracoidea				
Cicadellidae				
Cicadellinae	Cicadellini	*Bothrogonia ferruginea*	KU167550	Yu et al. (2019)
		*Bothrogonia yunana*	MT500858	Xu et al. (2021)
Coelidiinae	Coelidiini	*Olidiana obliquea*	MN780583	Wang, Wang, and Dai (2021)
		*Taharana fasciana*	KY886913	Wang, Li, and Dai (2017)
Deltocephalinae	Athysanini	*Abrus daozhenensis*	MZ274046	GenBank
		*Abrus expansivus*	MK033020	Wang and Xing (2019)
		*Abrus yunshanensis*	MZ274047	GenBank
		*Norvellina* sp.	KY039131	Song, Cai, and Li (2017)
		*Tambocerus* sp.	KT827824	Yu et al. (2017)
	Chiasmini	*Exitianus indicus*	KY039128	Song, Cai, and Li (2017)
		*Nephotettix cincticeps*	KP749836	GenBank
	Cicadulini	*Watanabella graminea*	MK234840	Yang et al. (2019)
	Deltocephalini	*Alobaldia tobae*	KY039116	Song, Cai, and Li (2017)
		*Deltocephalus uncinatus*	OP978288	This study
		*Deltocephalus vulgaris*	OP978291	This study
		*Deltocephalus vulgaris*	OP978289	This study

		*Graminella nigrinota*	OP978290	This study
		*Maiestas distincta*	OP972719	This study
		*Maiestas dorsalis*	KX786285	Du et al. (2017)
		*Maiestas heuksandoensis*	OP972720	This study
		*Maiestas horvathi*	OP972721	This study
		*Maiestas latifrons*	OP972722	This study
		*Maiestas obongsanensis*	OP972723	This study
		*Maiestas oryzae*	OP972724	This study
		*Maiestas remigia*	OP972725	This study
		*Maiestas subviridis*	OP972726	This study
		*Maiestas tareni*	OP972727	This study
		*Maiestas yangae*	OP972728	This study
	*Polyamia penistenuis*	OP972729	This study

	Drabescini	*Drabescoides nuchalis*	KR349344	Wu et al. (2016)
		*Drabescus ineffectus*	MT527188	Xu, Yu, and Zhang (2020)
	*Roxasellana stellata*	MT527187	Xu, Yu, and Zhang (2020)
Deltocephalinae	Fieberiellini	*Fieberiella septentrionalis*	MW078430	Luo et al. (2021)
	Macrostelini	*Macrosteles quadrilineatus*	KY645960	Mao, Yang, and Bennett (2017)
		*Macrosteles quadrimaculatus*	MG727894	Du, Dietrich, and Dai (2019)
	Mukariini	*Mukaria splendida*	MG813485	Yang and Dai (2021)
	Opsiini	*Hishimonoides recurvatis*	KY364883	GenBank
		*Japananus hyalinus*	KY129954	Du et al. (2017)
		*Orosius orientalis*	KY039146	Song, Cai, and Li (2017)
	Paralimnini	*Paralaevicephalus gracilipenis*	MK450366	Xing and Wang (2019)
		*Yanocephalus yanonis*	KY039113	Song, Cai, and Li (2017)
	Penthimiini	*Penthimia melanocephala*	MT768010	Xu and Dai (2021)

		*Reticuluma hamata*	MN922303	Xu and Dai (2020)
	Scaphoideini	*Changbaninus pleiospicules*	MT332317	GenBank
		*Scaphoideus maai*	KY817243	Du, Dai, and Dietrich (2017)
		*Scaphoideus maculatus*	MT332316	GenBank
		*Scaphoideus nigrivalveus*	KY817244	Du, Dai, and Dietrich (2017)
	*Scaphoideus varius*	KY817245	Du, Dai, and Dietrich (2017)
Eurymelinae	Idiocerini	*Populicerus confusus*	MT341642	Shan et al. (2020)
		*Populicerus populi*	MH492318	Wang et al. (2018)
Evacanthinae	Evacanthini	*Evacanthus danmainus*	MN227166	Du et al. (2021)
		*Evacanthus heimianus*	MN227167	Du et al. (2021)
Hylicinae	Hylicini	*Hylica paradoxa*	MW218660	Tang, Huang, and Zhang (2020)
	Sudrini	*Kalasha nativa*	MW218662	Tang, Huang, and Zhang (2020)
Iassinae	Batracomorphini	*Batracomorphus lateprocessus*	MG813489	Wang et al. (2020)
	Krisnini	*Krisna rufimarginata*	MN577636	Wang et al. (2020)
Ledrinae	Ledrini	*Petalocephala eurglobata*	MW018817	Wang et al. (2022)
		*Petalocephala gongshanensis*	MW018818	Wang et al. (2022)
Megophthalminae	Agalliini	*Durgades nigropicta*	KY123686	Wang et al. (2017)
		*Japanagallia spinosa*	KY123687	Wang et al. (2017)
Mileewinae	Mileewini	*Mileewa albovittata*	MK138358	He, Li, and Yang (2019)
		*Ujna puerana*	MZ326688	Yu and Zhang (2021)
Typhlocybinae	Empoascini	*Empoasca vitis*	KJ815009	Zhou et al. (2016)
		*Matsumurasca onukii*	MG190360	Liu et al. (2017)
Membracidae				
Centrotinae	Leptobelini	*Leptobelus gazella*	JF801955	Zhao and Liang (2016)
Smiliinae	Polyglyptini	*Entylia carinata*	KX495488	Mao, Yang, and Bennett (2016)

**FIGURE 8:**
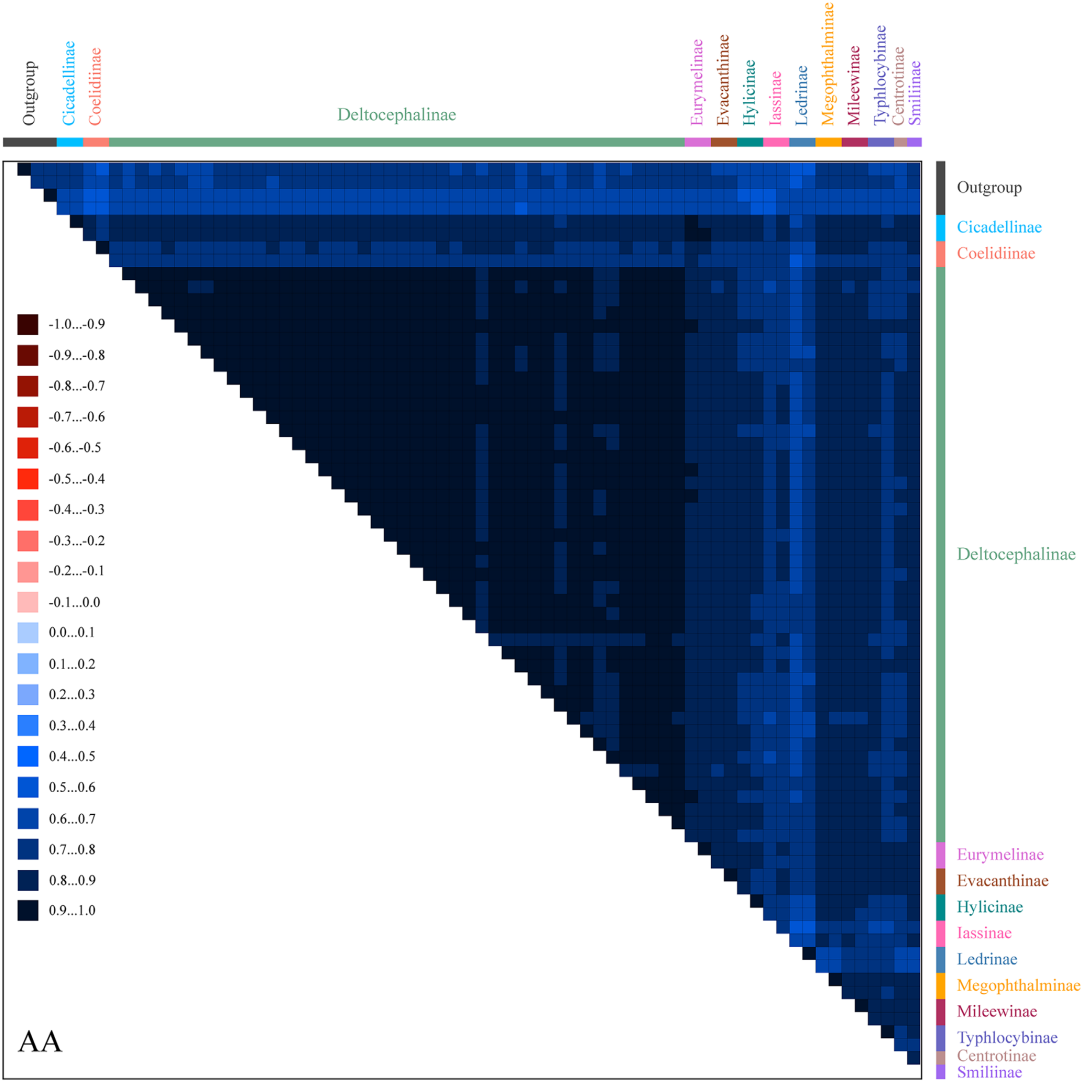
Heterogeneous sequence divergence within AA. The mean similarity score between sequences is represented by a colored square. AliGROOVE scores ranging from −1 (indicating great difference in rates from the remainder of the dataset; red coloring shows the significant heterogeneity) to +1 (indicating rates match in all other comparisons).

**FIGURE 9:**
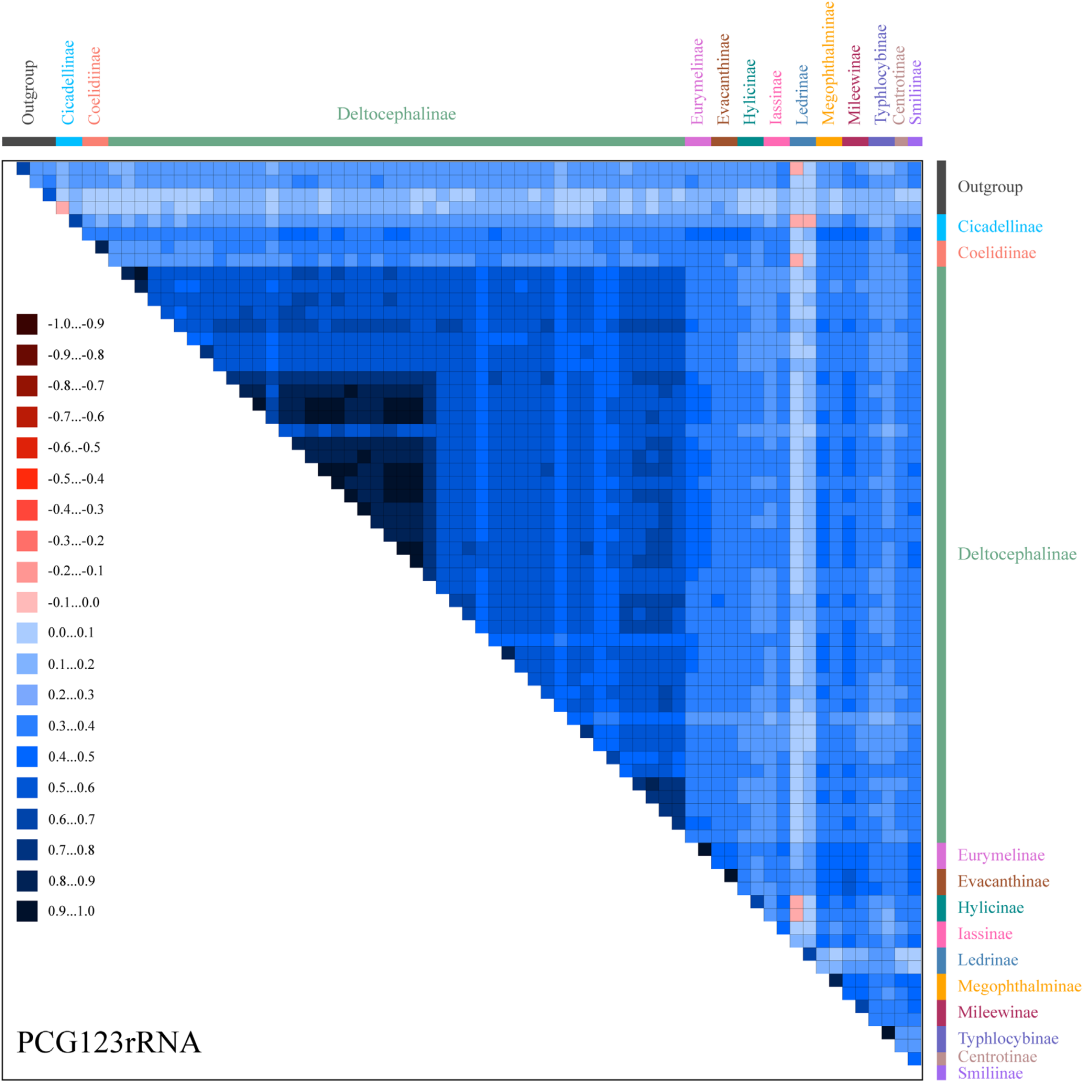
Heterogeneous sequence divergence within PCG123rRNA. The mean similarity score between sequences is represented by a colored square. AliGROOVE scores ranging from −1 (indicating great difference in rates from the remainder of the dataset; red coloring shows the significant heterogeneity) to +1 (indicating rates match in all other comparisons).

**FIGURE 10:**
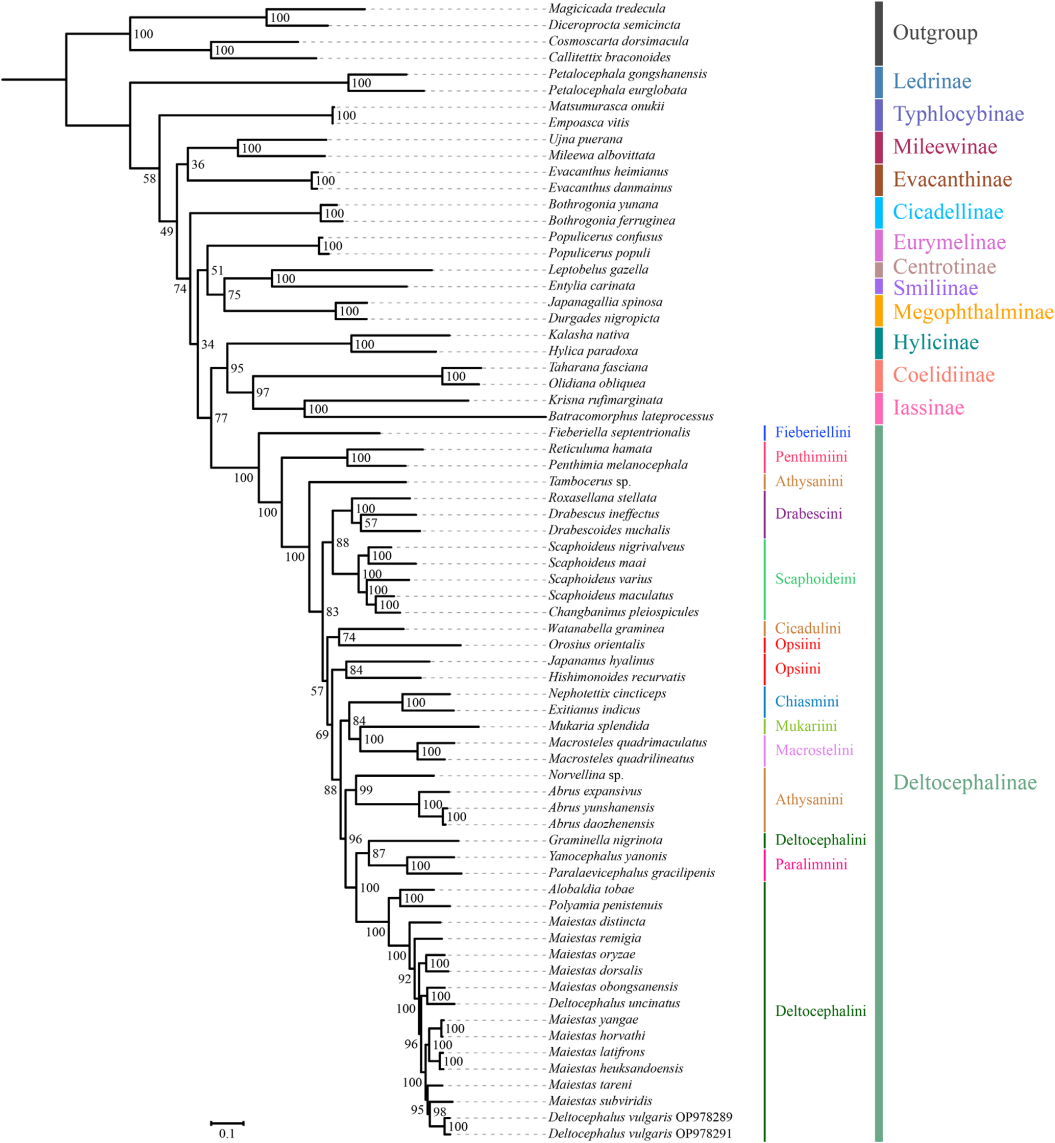
Phylogenetic relationships assessed using the maximum likelihood (ML) method based on the AA dataset. Numbers at each node correspond to the bootstrap values.

**FIGURE 11:**
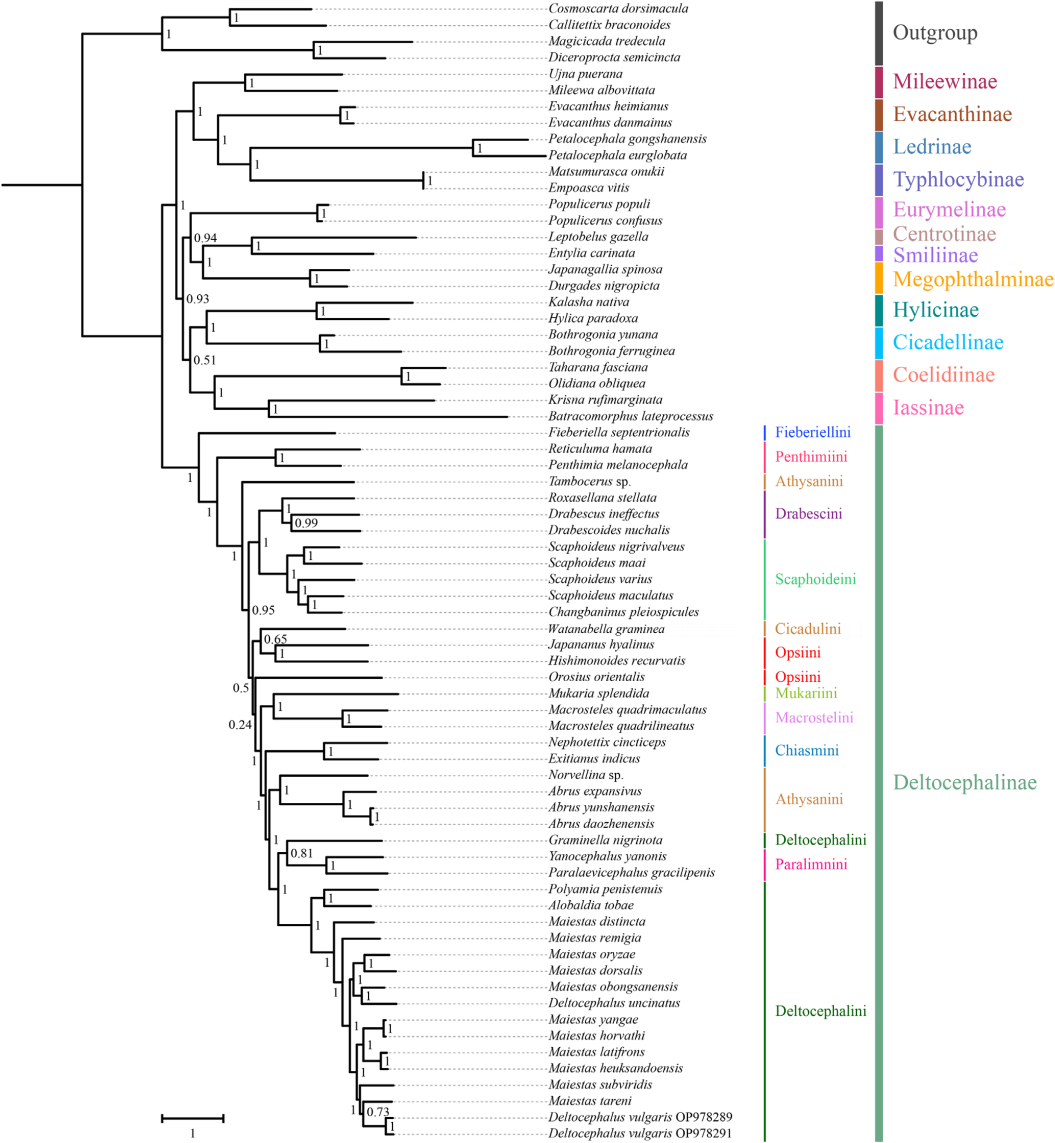
Phylogenetic relationships assessed using the Bayesian inference (BI) method based on the PCG123rRNA dataset. Numbers at each node correspond to the posterior probability (PP) values.

Mitogenome data of the 69 species were concatenated into five datasets: 13 PCGs with two rRNA sequences (PCG123rRNA), 13 protein‐coding gene sequences without rRNAs (PCG123), 13 PCG sequences with the third codon position removed plus two rRNAs (PCG12rRNA), 13 PCG sequences with the third codon position removed without rRNAs (PCG12), and amino acid sequences of 13 PCGs (AA). Tests of base substitution saturation indicated that the selected datasets were not saturated and therefore suitable for phylogenetic analysis. The analysis of the heterogeneity of the sequence divergence indicated that sequence divergence within each dataset was not significant, except Ledrinae in the PCG123rRNA and PCG123 datasets, and Cicadellinae in the PCG123rRNA dataset. Removing the third codon position from the datasets reduced heterogeneity (Figures 8 and 9; Figures S16–, S18). Maximum likelihood (ML) and Bayesian inference (BI) methods were used for phylogenetic analysis. Based on the 10 phylogenetic trees obtained from the ML and BI analyses of the five datasets (Figures 10 and 11; Figures S19–, S26), the representatives of each subfamily for which more than one representative was included (Coelidiinae, Deltocephalinae, Eurymelinae, Evacanthinae, Hylicinae, Iassinae, Ledrinae, Megophthalminae, Mileewinae, and Typhlocybinae) formed monophyletic groups (bootstrap values [BS] = 100; posterior probability [PP] = 1.0). Similar to other recent molecular phylogenetic analyses, branches pertaining to the relationships among leafhopper subfamilies received only low‐to‐moderate support and were unstable in analyses. Ledrinae was the earliest diverging cicadellid subfamily in the AA‐ML tree (Figure 10). Mileewinae was the earliest diverging cicadellid subfamily in both the PCG123rRNA‐ML and PCG12rRNA‐ML trees. The remaining seven phylogenetic trees placed Deltocephalinae as a sister taxon to the remaining subfamilies (Figure 11; Figures S19–, S26). In the PCG12rRNA‐ML and AA‐ML trees, Deltocephalinae was sister to (Hylicinae + (Coelidiinae + Iassinae)), but this branch received low‐to‐medium support in two trees (PCG12rRNA–ML–BS = 56, AA–ML–BS = 77). In the PCG12rRNA‐ML tree, ((Hylicinae + Cicadellinae) + (Coelidiina*e* + Iassinae)) was sister to Deltocephalinae; however, this branch also had very low support (PCG123rRNA–ML–BS = 31).

Within Deltocephalinae, phylogenies based on mitogenome sequence data were consistent with the recent anchored‐hybrid‐based phylogeny of Cao et al. (2022). In all phylogenetic trees, 11 clades of the subfamily were monophyletic; however, tribes Athysanini, Opsiini, and Deltocephalini were not consistently recovered. Deltocephalini and Paralimnini consistently clustered together and exhibited high support for sister‐group relationships (BS = 100; PP = 1). Only in the PCG123‐ML tree, tribe Opsiini formed a single clade but with low support.

In all phylogenetic trees, *Graminella nigrinota* did not group with other Deltocephalini, but rather with Paralimnini, with medium‐to‐high levels of support (BS > 87; PP > 0.81). Except for *Graminella nigrinota*, the remaining 4 genera and 15 species of tribe Deltocephalini formed well‐supported monophyletic groups (BS = 100, PP = 1). Within *Deltocephalus*, two morphological variants of *Deltocephalus vulgaris* clustered strongly into single units (BS = 100; PP = 1). The phylogenetic results obtained from the two different analytical methods did not vary significantly, and relationships within this clade were well‐resolved under a homogenous substitution model. Therefore, we performed phylogenetic analysis under the homogenous model only.

Here, 10 phylogenetic trees constructed based on the five datasets and two analysis methods (Figures 10, 11; Figures S19–, S26) revealed that the mitochondrial genome sequences indicate the relationships within and among the tribes of deltocephaline leafhoppers and within tribe Deltocephalini (BS = 100; PP = 1.0). Within Deltocephalini (except for *Graminella nigrinota*), the topology was divided into two major branches, (*Alobaldia tobae* + *Polyamia penistenuis*) forming a well‐supported sister clade with the remaining species of the tribe. The topology shows that the branch composed of the included species of the genus *Maiestas* is paraphyletic relative to the single representatives of the genera *Alobaldia* and *Polyamia*, forming a structure of ((*Alobaldia* + *Polyamia*) + *Maiestas*). In the PCG123rRNA‐BI, AA‐ML, and AA‐BL trees, differences were observed in the relationships between some species of *Maiestas* and *Deltocephalus*, with neither genus being recovered as monophyletic. In the remaining seven phylogenetic trees, the topologies of *Maiestas* were: (*Maiestas distincta* + (*Maiestas remigia* + (((*Maiestas oryzae* + *Maiestas dorsalis*) + (*Maiestas obongsanensis* + *Deltocephalus uncinatus*)) + (((*Maiestas yangae* + *Maiestas horvathi*) + (*Maiestas latifrons* + *Maiestas heuksandoensis*)) + (*Maiestas tareni* + (*Maiestas subviridis* + *Deltocephalus vulgaris*)))))). In contrast, in the AA‐ML and AA‐BI trees, the topologies were: (*Maiestas distincta* + (*Maiestas remigia* + ((*Maiestas oryzae* + *Maiestas dorsalis*) + ((*Maiestas obongsanensis* + *Deltocephalus uncinatus*) + (((*Maiestas yangae* + *Maiestas horvathi*) + (*Maiestas latifrons* + *Maiestas heuksandoensis*)) + (*Maiestas tareni* + (*Maiestas subviridis* + *Deltocephalus vulgaris*))))))). The main difference between these two topologies lies in the positions of the (*Maiestas oryzae* + *Maiestas dorsalis*) branches. Based on the branch support, phylogenetic relationships within Deltocephalini based on the mitochondrial PCG123rRNA‐ML, PCG123‐ML, PCG12rRNA‐ML, PCG12‐ML, PCG123‐BI, PCG12rRNA‐BI, and PCG12‐BI tree datasets were found to be reliable. All analyses yielded results largely consistent with those of Cao et al. (2022) and Wu et al. (2023).

## 4 Conclusions

Comparative analysis of the complete mitogenomes of 16 species representing five genera of leafhopper tribe Deltocephalini revealed a highly conserved overall genome structure and composition in this group, without any of the gene rearrangements reported for other groups of deltocephaline leafhoppers. Overall, our phylogenetic results suggest that analyses of mitogenome sequence data provide good resolution of deep and shallow branches of the phylogeny of Deltocephalini and other leafhoppers and yield results similar to those of the analyses of nuclear genes alone or combined nuclear and mitochondrial genes. However, the currently available mitogenome sequences only account for a small fraction of the known species. Therefore, further sequencing efforts are necessary to increase the taxon sample and facilitate further exploration of leafhopper phylogeny.

## Author Contributions

**Bingqing Xie:** data curation (equal), formal analysis (equal), investigation (equal), methodology (equal), software (equal), writing – original draft (equal), writing – review and editing (equal). **Xinyi Zhang:** data curation (equal), formal analysis (equal), investigation (equal), methodology (equal), writing – review and editing (equal). **Yongxia Zhang:** data curation (equal), methodology (equal), software (equal), writing – original draft (equal), writing – review and editing (equal). **Christopher H. Dietrich:** conceptualization (equal), writing – review and editing (equal). **Yani Duan:** conceptualization (equal), data curation (equal), funding acquisition (lead), project administration (lead), resources (lead), supervision (lead), writing – review and editing (equal).

## Conflicts of Interest

The authors declare no conflicts of interest.

## Supporting information


**Figure S1.** Predicted secondary structures of the transfer RNA (tRNA) genes of *Deltocephalus uncinatus*.

**Figure S2.** Predicted secondary structures of the tRNA genes of *Deltocephalus vulgaris* (OP978289).

**Figure S3.** Predicted secondary structures of the tRNA genes of *Deltocephalus vulgaris* (OP978291).

**Figure S4.** Predicted secondary structures of the tRNA genes of *Graminella nigrinota*.

**Figure S5.** Predicted secondary structures of the tRNA genes of *Maiestas distincta*.

**Figure S6.** Predicted secondary structures of the tRNA genes of *Maiestas heuksandoensis*.

**Figure S7.** Predicted secondary structures of the tRNA genes of *Maiestas horvathi*.

**Figure S8.** Predicted secondary structures of the tRNA genes of *Maiestas latifrons*.

**Figure S9.** Predicted secondary structures of the tRNA genes of *Maiestas obongsanensis*.

**Figure S10.** Predicted secondary structures of the tRNA genes of *Maiestas oryzae*.

**Figure S11.** Predicted secondary structures of the tRNA genes of *Maiestas remigia*.

**Figure S12.** Predicted secondary structures of the tRNA genes of *Maiestas subviridis*.

**Figure S13.** Predicted secondary structures of the tRNA genes of *Maiestas tareni*.

**Figure S14.** Predicted secondary structures of the tRNA genes of *Maiestas yangae*.

**Figure S15.** Predicted secondary structures of the tRNA genes of *P. penistenuis*.

**Figure S16.** Heterogeneous sequence divergence within PCG12.

**Figure S17.** Heterogeneous sequence divergence within PCG12rRNA.

**Figure S18.** Heterogeneous sequence divergence within PCG123.

**Figure S19.** Phylogenetic relationships assessed using the Bayesian inference (BI) method based on the AA dataset. Numbers at each node correspond to the posterior probability (PP) values.

**Figure S20.** Phylogenetic relationships assessed using the BI method based on the PCG12 dataset. Numbers at each node correspond to the PP values.

**Figure S21.** Phylogenetic relationships assessed using the maximum likelihood (ML) method based on the PCG12 dataset. Numbers at each node correspond to the bootstrap values.

**Figure S22.** Phylogenetic relationships assessed using the BI method based on the PCG12rRNA dataset. Numbers at each node correspond to the PP values.

**Figure S23.** Phylogenetic relationships assessed using the ML method based on the PCG12rRNA dataset. Numbers at each node correspond to the bootstrap values.

**Figure S24.** Phylogenetic relationships assessed using the BI method based on the PCG123 dataset. Numbers at each node correspond to the PP values.

**Figure S25.** Phylogenetic relationships assessed using the ML method based on the PCG123 dataset. Numbers at each node correspond to the bootstrap values.

**Figure S26.** Phylogenetic relationships assessed using the ML method based on the PCG123rRNA dataset. Numbers at each node correspond to the bootstrap values.

**Table S1.** Best partitioning scheme and models for different datasets selected using PartitionFinder.

## Data Availability

Mitogenome sequences of Deltocephalini have been deposited in GenBank, and the assigned accession numbers are listed Table 1.
