# Supplementary figures and images for: Comparative Analysis of Mitogenomes in Leafhopper Tribe Deltocephalini (Hemiptera: Cicadellidae: Deltocephalinae): Structural Conservatism and Phylogeny
Source: Ecol Evol. 2024 Dec 18;14(12):e70738. doi: 10.1002/ece3.70738 (PMC11655181; doi:10.1002/ece3.70738)

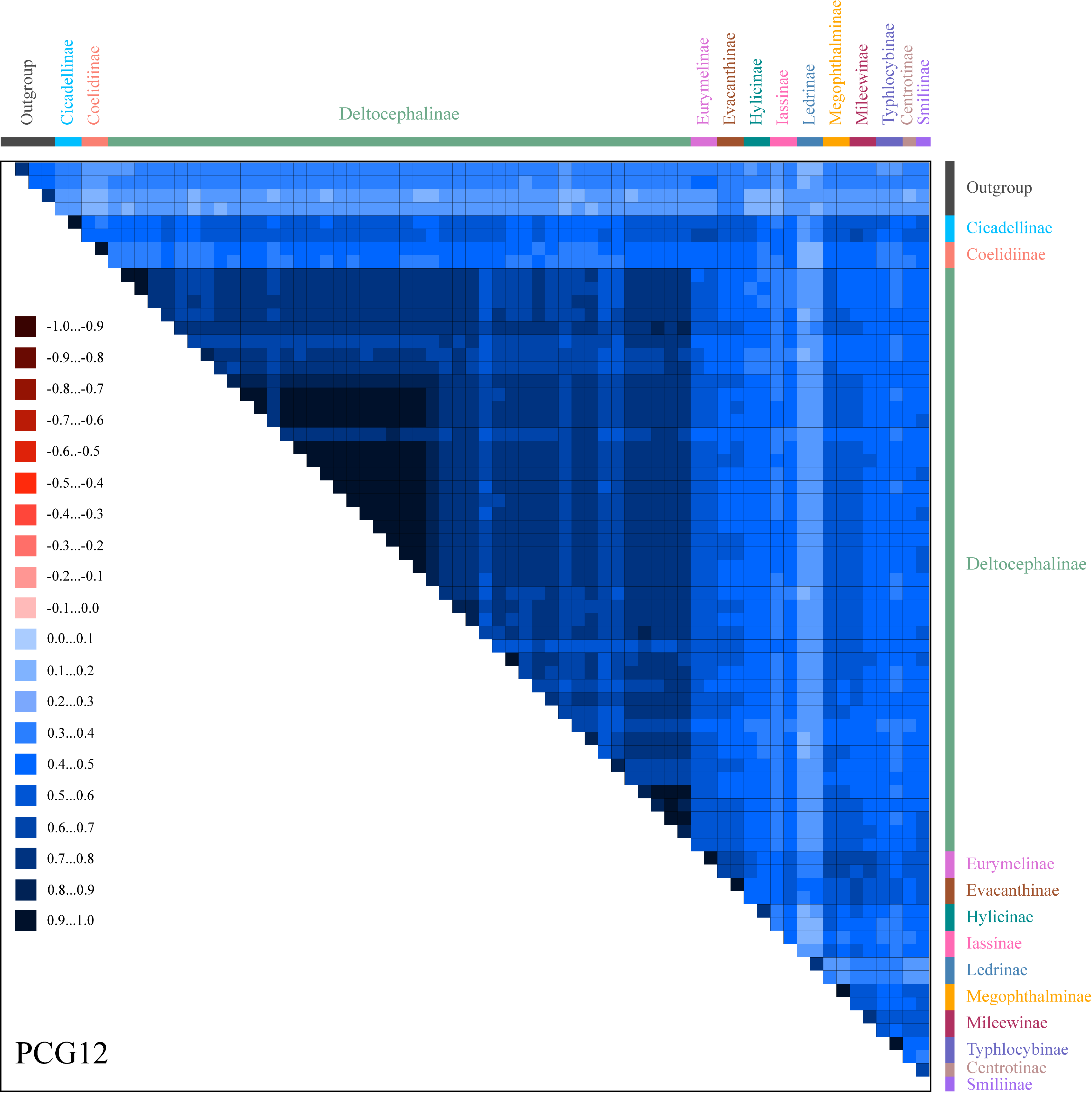

Supplement: Supplementary file 16 — Figure S16. Heterogeneous sequence divergence within PCG12. [file ECE3-14-e70738-s011.tif]

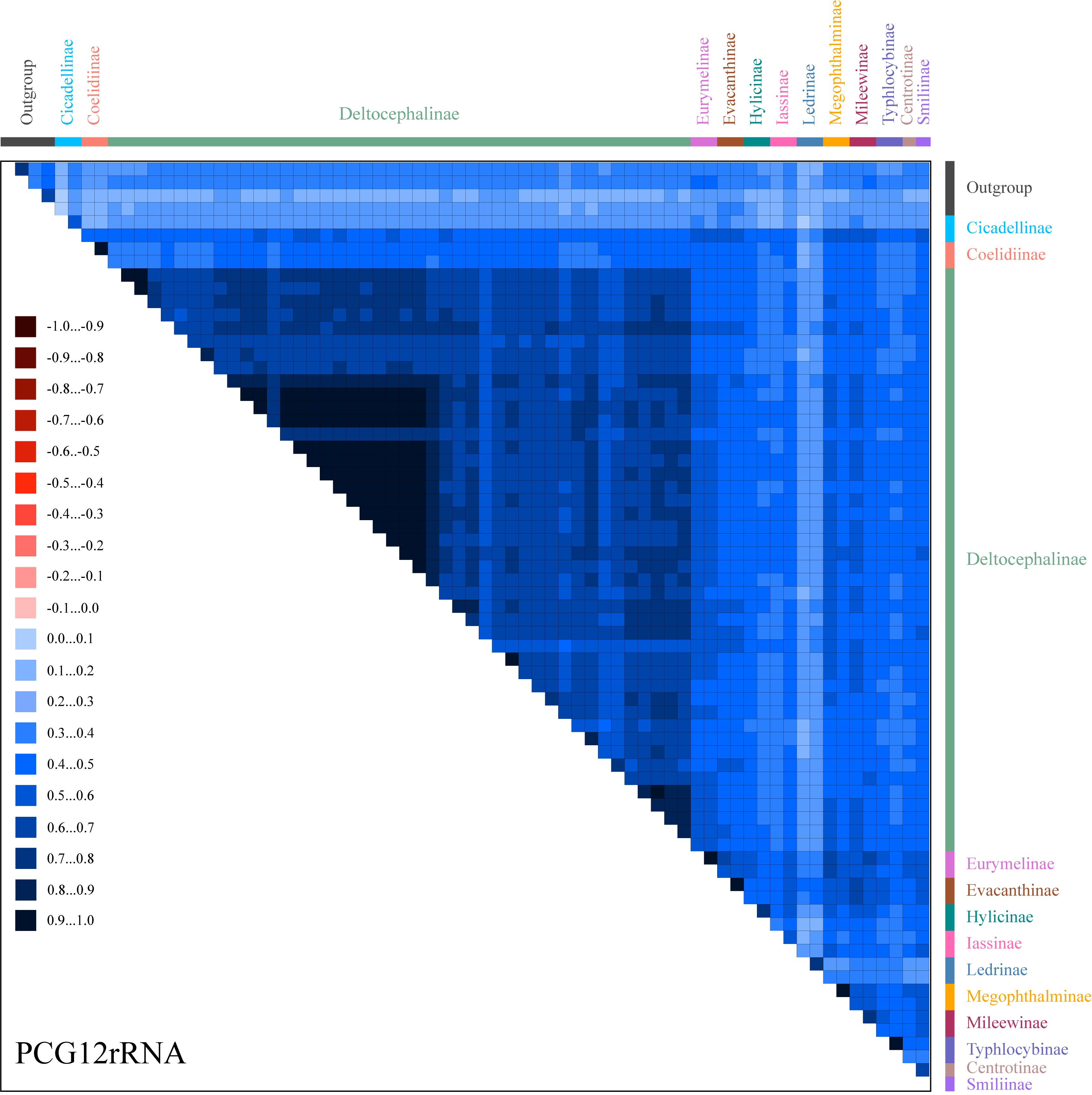

Supplement: Supplementary file 17 — Figure S17. Heterogeneous sequence divergence within PCG12rRNA. [file ECE3-14-e70738-s025.tif]

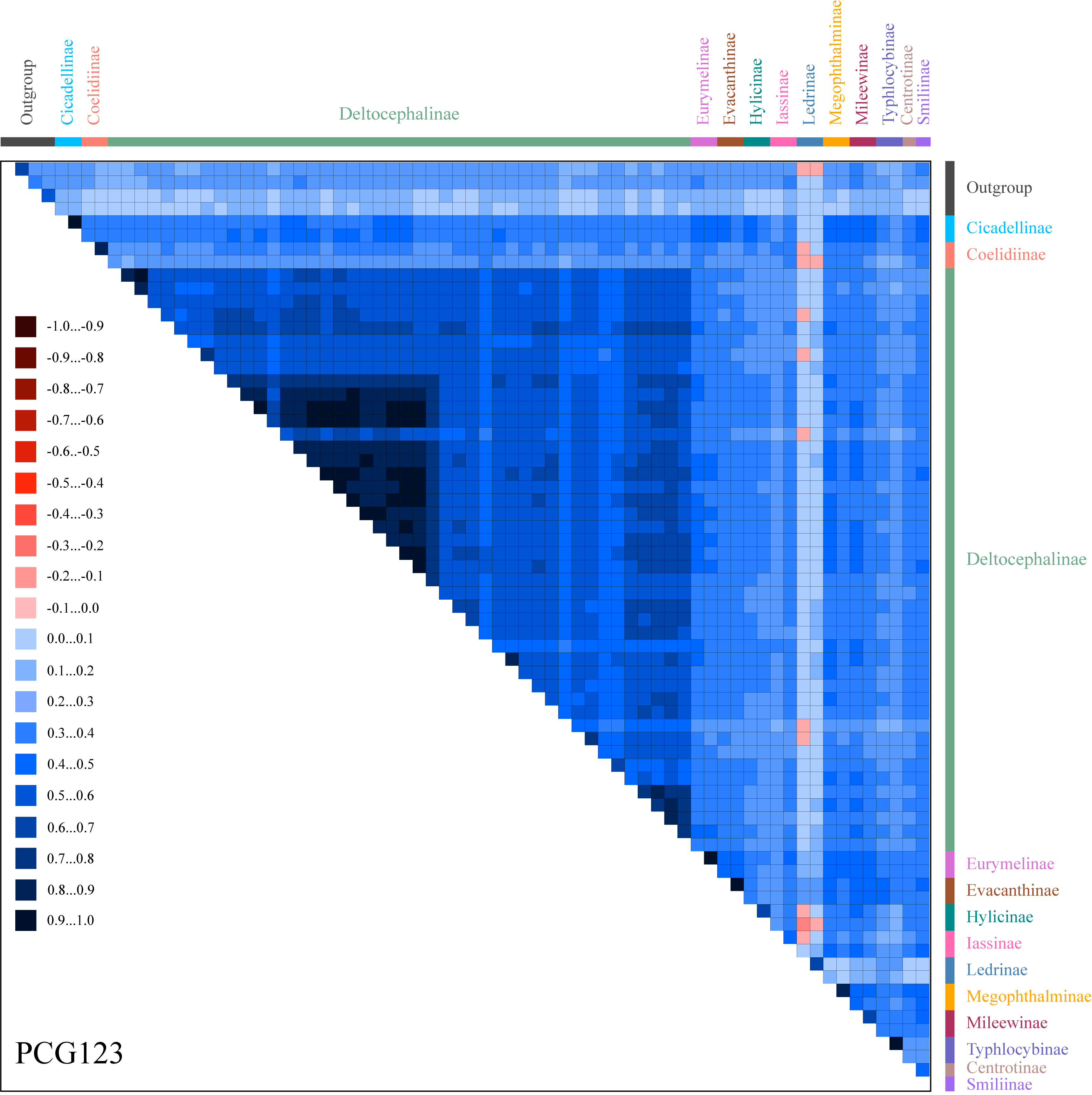

Supplement: Supplementary file 18 — Figure S18. Heterogeneous sequence divergence within PCG123. [file ECE3-14-e70738-s002.tif]

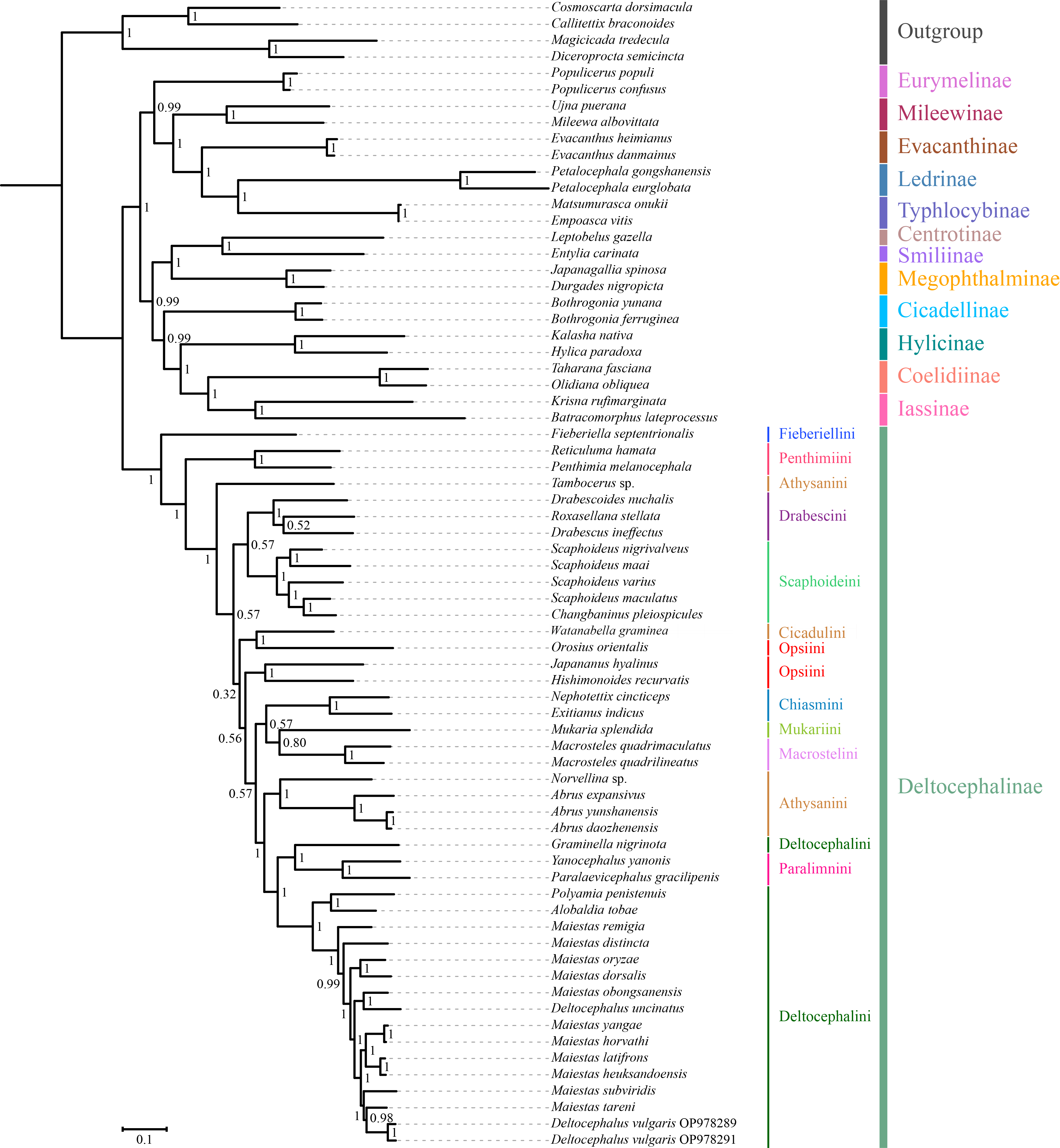

Supplement: Supplementary file 19 — Figure S19. Phylogenetic relationships assessed using the Bayesian inference (BI) method based on the AA dataset. Numbers at each node correspond to the posterior probability (PP) values. [file ECE3-14-e70738-s019.tif]

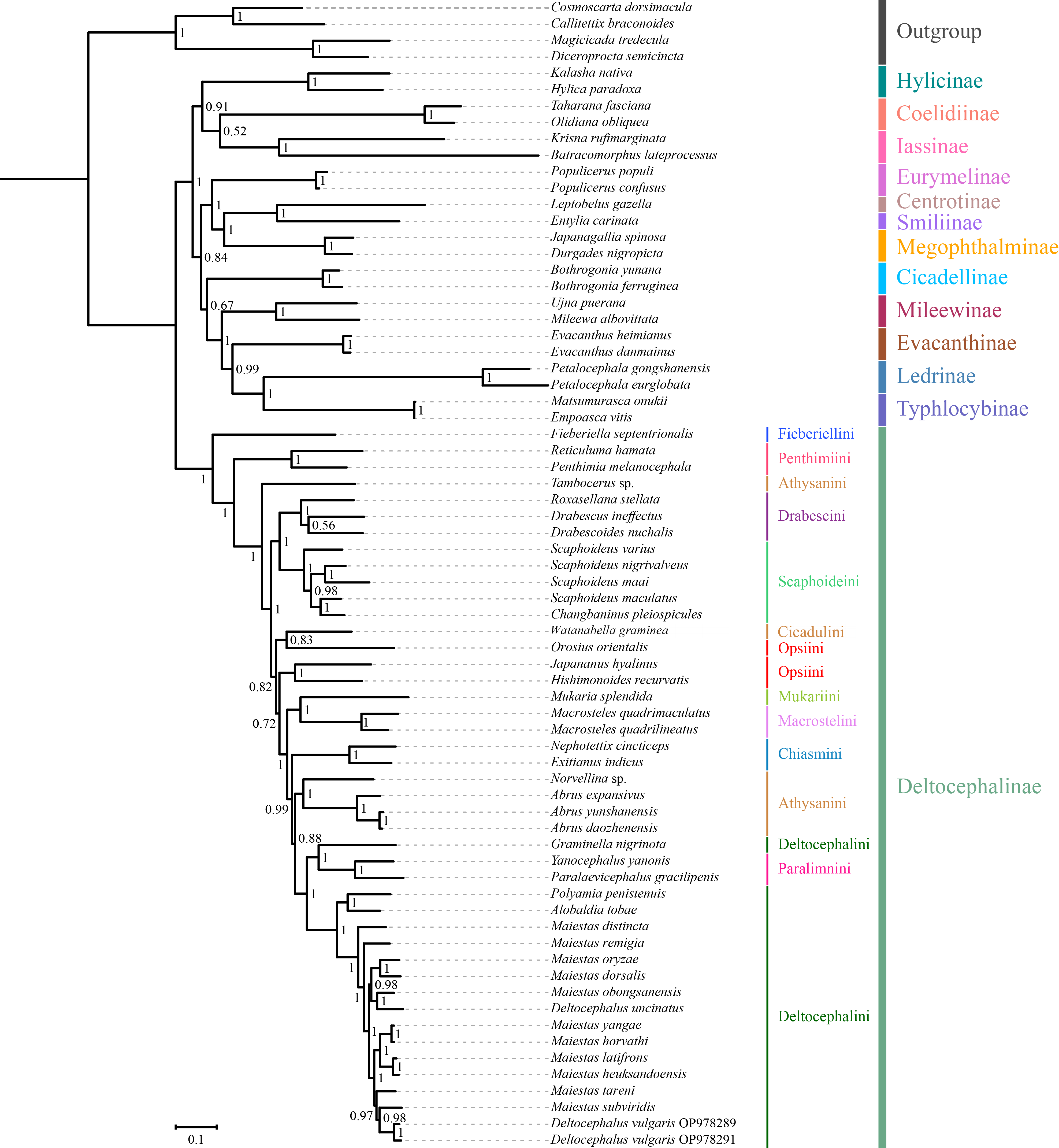

Supplement: Supplementary file 20 — Figure S20. Phylogenetic relationships assessed using the BI method based on the PCG12 dataset. Numbers at each node correspond to the PP values. [file ECE3-14-e70738-s010.tif]

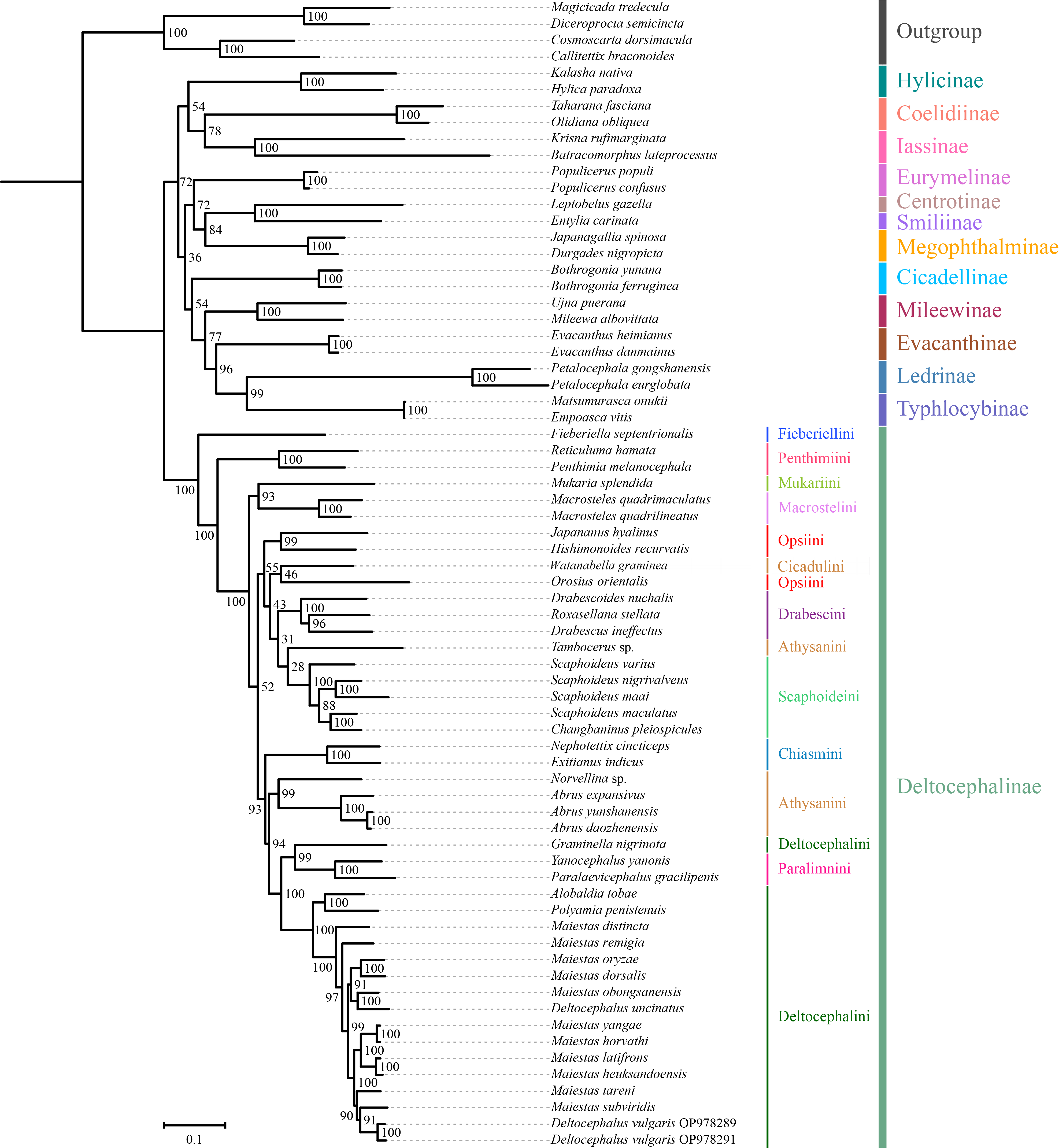

Supplement: Supplementary file 21 — Figure S21. Phylogenetic relationships assessed using the maximum likelihood (ML) method based on the PCG12 dataset. Numbers at each node correspond to the bootstrap values. [file ECE3-14-e70738-s024.tif]

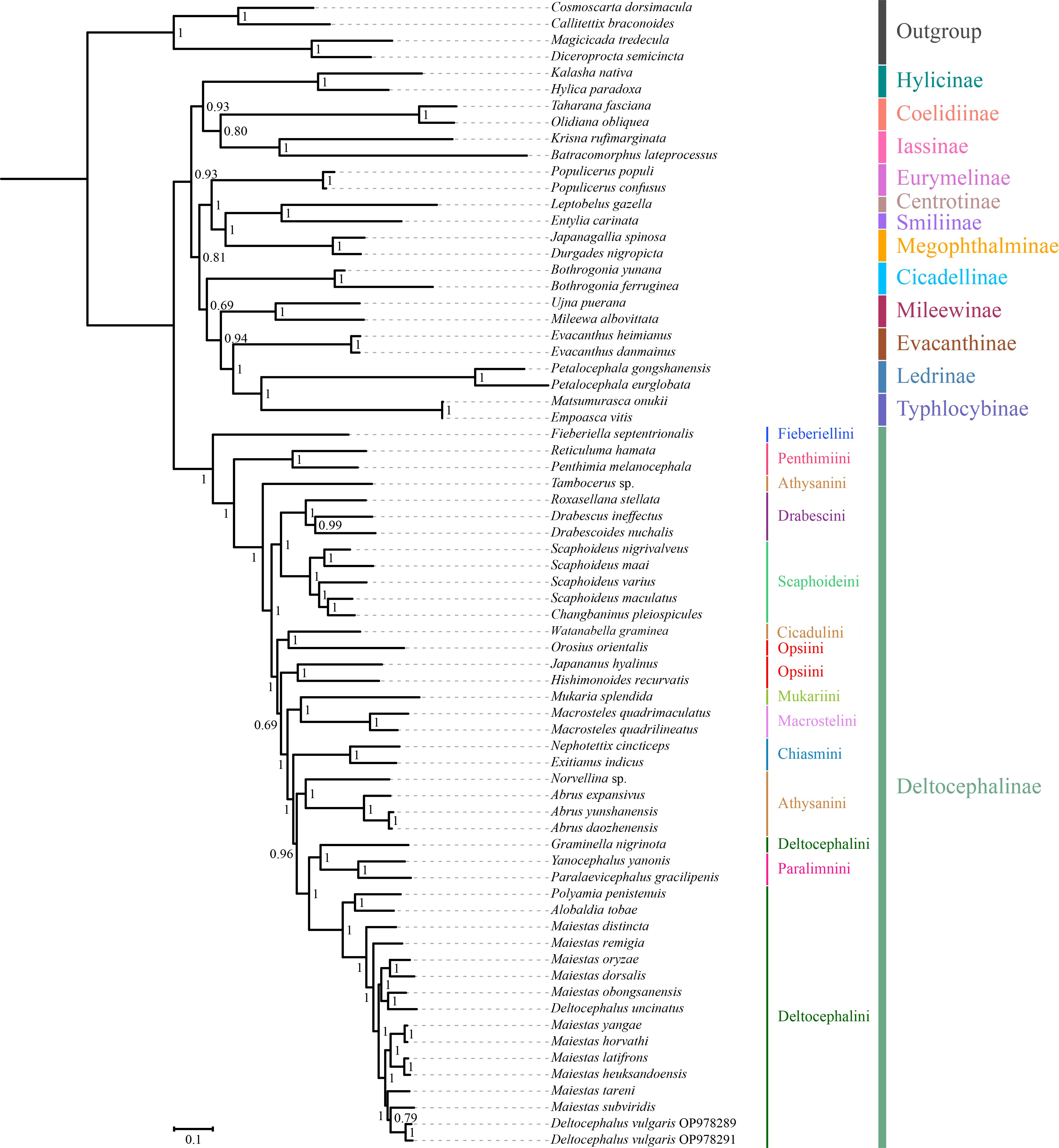

Supplement: Supplementary file 22 — Figure S22. Phylogenetic relationships assessed using the BI method based on the PCG12rRNA dataset. Numbers at each node correspond to the PP values. [file ECE3-14-e70738-s018.tif]

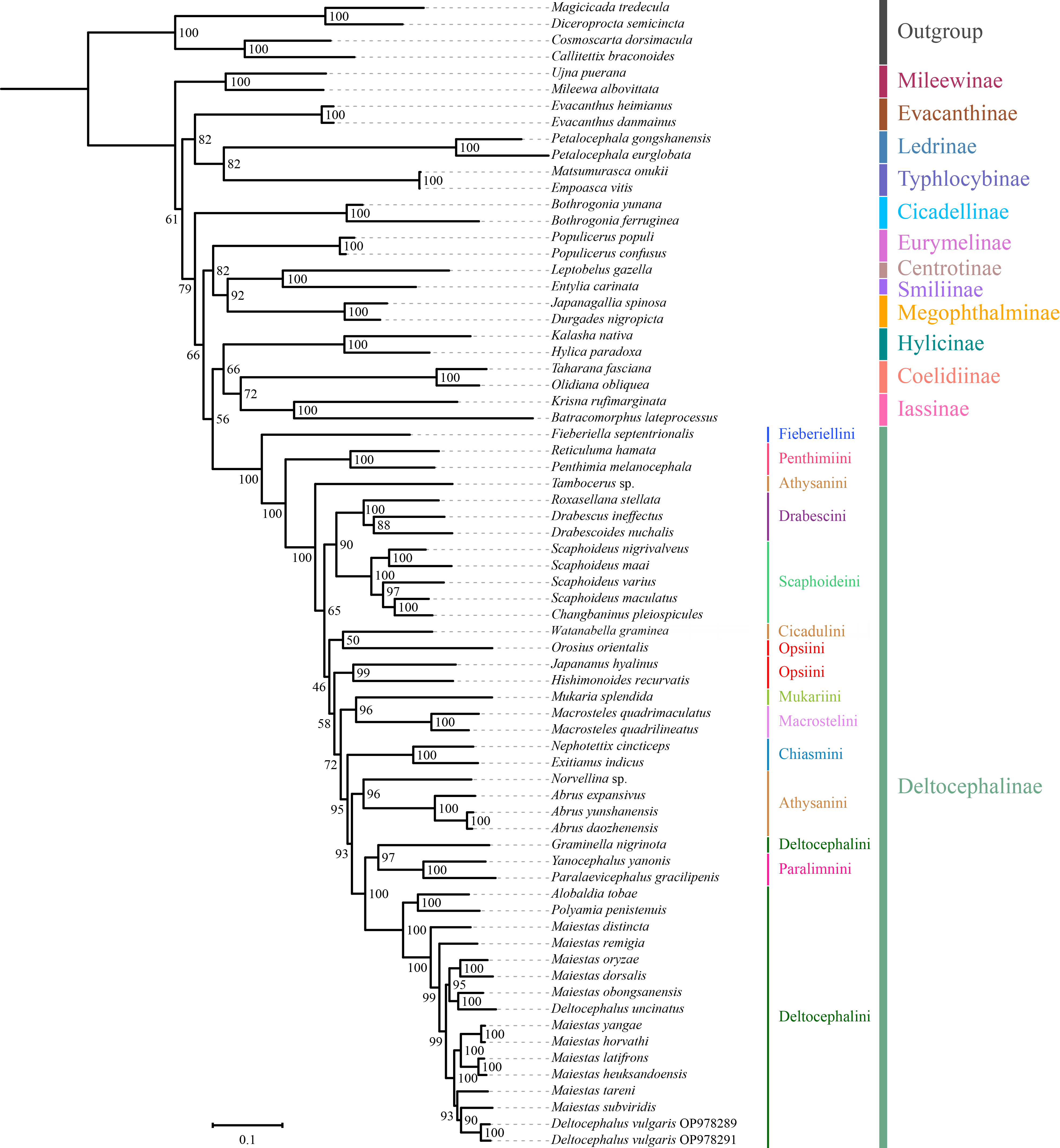

Supplement: Supplementary file 23 — Figure S23. Phylogenetic relationships assessed using the ML method based on the PCG12rRNA dataset. Numbers at each node correspond to the bootstrap values. [file ECE3-14-e70738-s004.tif]

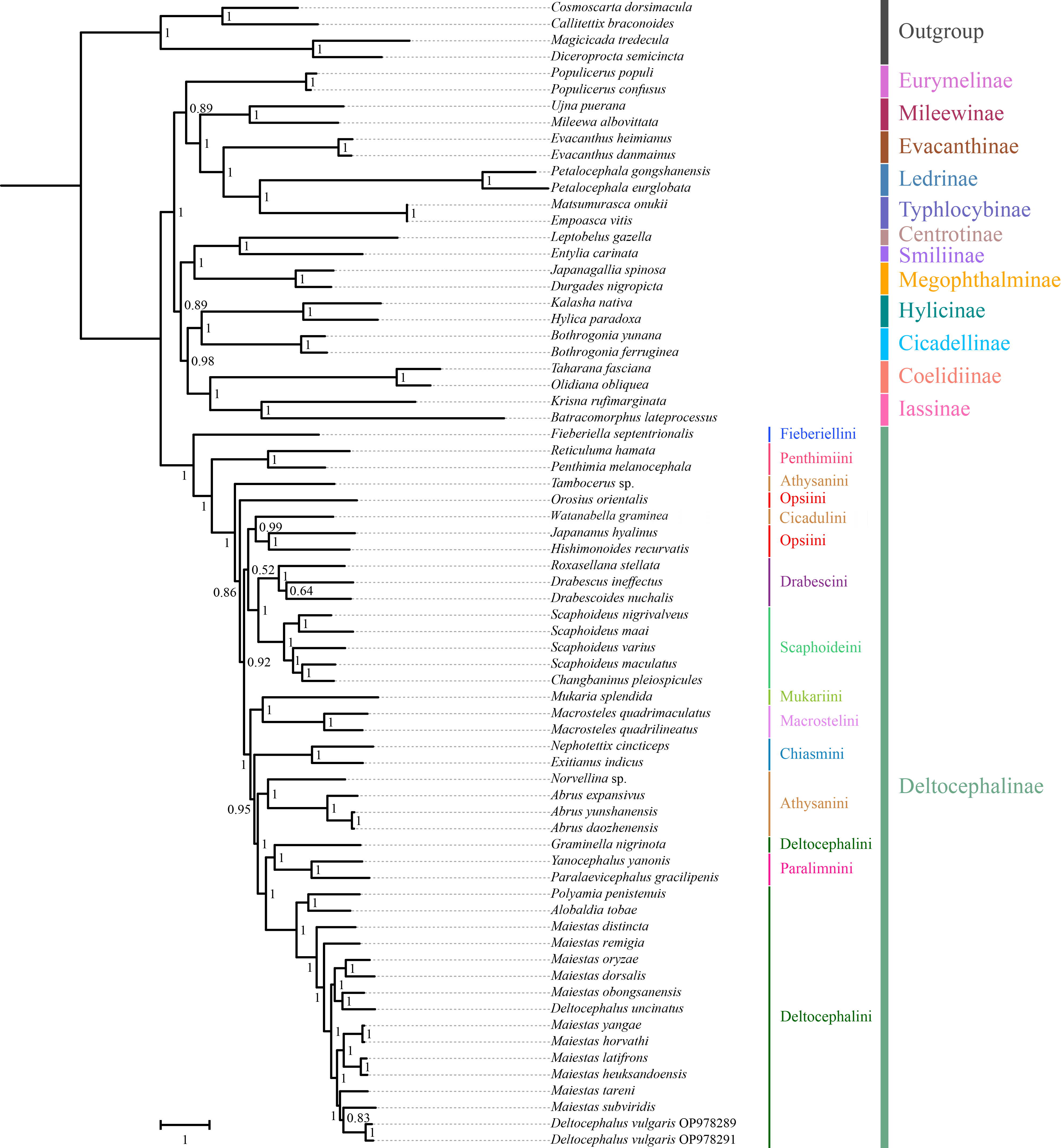

Supplement: Supplementary file 24 — Figure S24. Phylogenetic relationships assessed using the BI method based on the PCG123 dataset. Numbers at each node correspond to the PP values. [file ECE3-14-e70738-s001.tif]

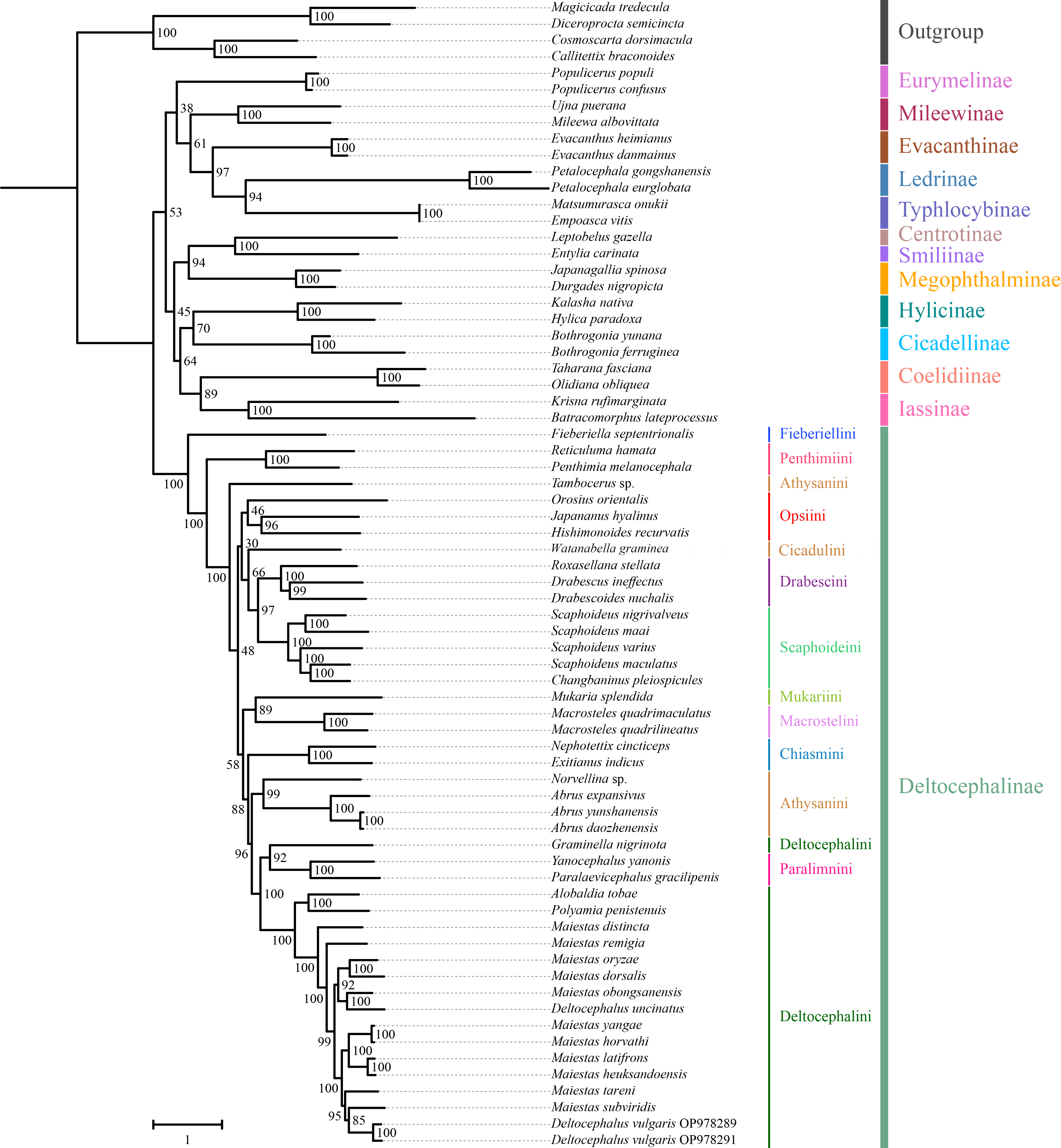

Supplement: Supplementary file 25 — Figure S25. Phylogenetic relationships assessed using the ML method based on the PCG123 dataset. Numbers at each node correspond to the bootstrap values. [file ECE3-14-e70738-s014.tif]

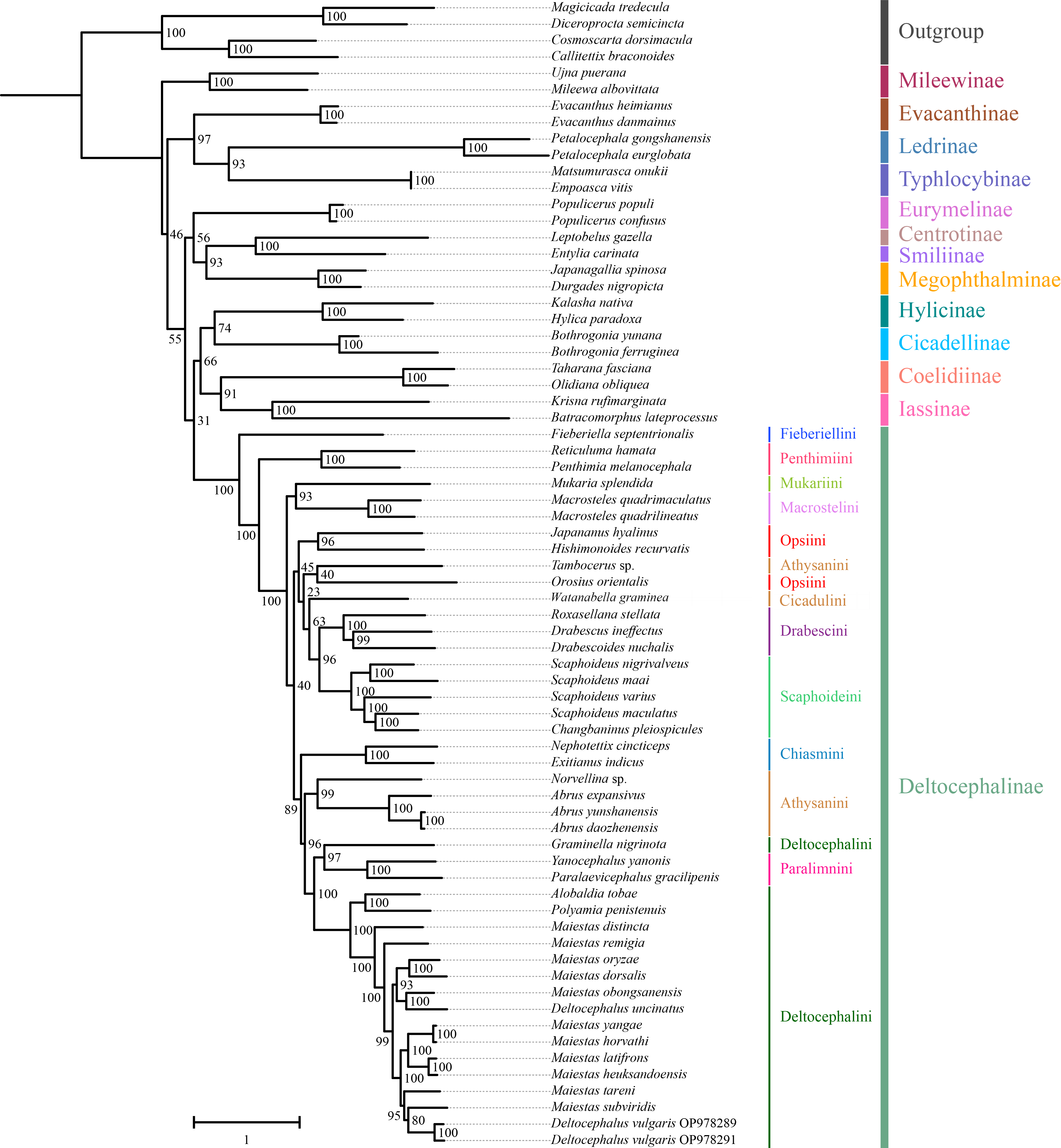

Supplement: Supplementary file 26 — Figure S26. Phylogenetic relationships assessed using the ML method based on the PCG123rRNA dataset. Numbers at each node correspond to the bootstrap values. [file ECE3-14-e70738-s021.tif]
