# Supplementary material for: Comparative Analysis of Mitogenomes in Leafhopper Tribe Deltocephalini (Hemiptera: Cicadellidae: Deltocephalinae): Structural Conservatism and Phylogeny
Source: Ecol Evol. 2024 Dec 18;14(12):e70738. doi: 10.1002/ece3.70738 (PMC11655181; doi:10.1002/ece3.70738)
Supplement: Supplementary file 27 — Table S1. Best partitioning scheme and models for different datasets selected using PartitionFinder. [file ECE3-14-e70738-s005.docx]

**Table S1:** Best partitioning scheme and models for different datasets selected by PartitionFinder.

| **Data matrix** | **Subset Partitions** | **Models** |
| --- | --- | --- |
| **PCG123rRNA–ML** | P1: *ATP6*_pos1, *CYTB*_pos1, *COX3*_pos1, *COX2*_pos1 | GTR+I+G |
|  | P2: *COX3*_pos2, *COX2*_pos2, *ATP6*_pos2 | TVM+I+G |
|  | P3: *COX3*_pos3, *ATP8*_pos3, *ATP6*_pos3, *ND6*_pos3, *ND3*_pos3 | K81UF+I+G |
|  | P4: *ND3*_pos1, *ND2*_pos1, *ATP8*_pos1, *ND6*_pos1 | GTR+I+G |
|  | P5: *ATP8*_pos2, *ND3*_pos2, *ND2*_pos2, *ND6*_pos2 | TVM+I+G |
|  | P6: *COX1*_pos1 | GTR+I+G |
|  | P7: *CYTB*_pos2, *COX1*_pos2 | TVM+I+G |
|  | P8: *CYTB*_pos3, *COX1*_pos3, *COX2*_pos3 | GTR+G |
|  | P9: *ND1*_pos1, *ND5*_pos1, *ND4L*_pos1, *ND4*_pos1 | GTR+I+G |
|  | P10: *ND1*_pos2, *ND5*_pos2, *ND4L*_pos2, *ND4*_pos2 | GTR+I+G |
|  | P11: *ND1*_pos3 | TIM+G |
|  | P12: *ND2*_pos3 | TRN+G |
|  | P13: *ND4*_pos3, *ND4L*_pos3, *ND5*_pos3 | HKY+G |
|  | P14: *rrnL*, *rrnS* | GTR+I+G |
| **PCG123rRNA–BI** | P1: *ATP6*_pos1, *CYTB*_pos1, *COX3*_pos1, *COX2*_pos1 | GTR+I+G |
|  | P2: *COX3*_pos2, *COX2*_pos2, *ATP6*_pos2 | GTR+I+G |
|  | P3: *COX3*_pos3, *ND6*_pos3, *ND3*_pos3, *ATP8*_pos3, *ATP6*_pos3 | GTR+I+G |
|  | P4: *ND3*_pos1, *ND2*_pos1, *ND6*_pos1, *ATP8*_pos1 | GTR+I+G |
|  | P5: *ATP8*_pos2, *ND3*_pos2, *ND6*_pos2, *ND2*_pos2 | GTR+I+G |
|  | P6: *COX1*_pos1 | GTR+I+G |
|  | P7: *CYTB*_pos2, *COX1*_pos2 | GTR+I+G |
|  | P8: *COX1*_pos3, *COX2*_pos3, *CYTB*_pos3 | GTR+G |
|  | P9: *ND5*_pos1, *ND4*_pos1, *ND1*_pos1, *ND4L*_pos1 | GTR+I+G |
|  | P10: *ND1*_pos2, *ND4*_pos2, *ND5*_pos2, *ND4L*_pos2 | GTR+I+G |
|  | P11: *ND1*_pos3 | GTR+G |
|  | P12: *ND2*_pos3 | GTR+G |
|  | P13: *ND5*_pos3, *ND4*_pos3, *ND4L*_pos3 | HKY+G |
|  | P14: *rrnL*, *rrnS* | GTR+I+G |
| **PCG123–ML** | P1: *ATP6*_pos1, *COX3*_pos1, *COX2*_pos1 | GTR+I+G |
|  | P2: *COX3*_pos2, *COX2*_pos2, *ATP6*_pos2 | TVM+I+G |
|  | P3: *COX3*_pos3, *ATP8*_pos3, *ATP6*_pos3, *ND6*_pos3, *ND3*_pos3 | K81UF+I+G |
|  | P4: *ND3*_pos1, *ND2*_pos1, *ATP8*_pos1, *ND6*_pos1 | GTR+I+G |
|  | P5: *ATP8*_pos2, *ND3*_pos2, *ND2*_pos2, *ND6*_pos2 | TVM+I+G |
|  | P6: *COX1*_pos1, *CYTB*_pos1 | GTR+I+G |
|  | P7: *CYTB*_pos2, *COX1*_pos2 | TVM+I+G |
|  | P8: *CYTB*_pos3, *COX1*_pos3, *COX2*_pos3 | GTR+G |
|  | P9: *ND1*_pos1, *ND5*_pos1, *ND4L*_pos1, *ND4*_pos1 | GTR+I+G |
|  | P10: *ND1*_pos2, *ND5*_pos2, *ND4L*_pos2, *ND4*_pos2 | GTR+I+G |
|  | P11: *ND1*_pos3 | TIM+G |
|  | P12: *ND2*_pos3 | TRN+G |

**Table S1.** *Cont.*

| **Data matrix** | **Subset Partitions** | **Models** |
| --- | --- | --- |
| **PCG123–ML** | P13: *ND4*_pos3, *ND4L*_pos3, *ND5*_pos3 | HKY+G |
| **PCG123–BI** | P1: *ATP6*_pos1, *COX3*_pos1, *COX2*_pos1 | GTR+I+G |
|  | P2: *COX3*_pos2, *COX2*_pos2, *ATP6*_pos2 | GTR+I+G |
|  | P3: *COX3*_pos3, *ND6*_pos3, *ND3*_pos3, *ATP8*_pos3, *ATP6*_pos3 | GTR+I+G |
|  | P4: *ATP8*_pos1, *ND3*_pos1, *ND6*_pos1, *ND2*_pos1 | GTR+I+G |
|  | P5: *ATP8*_pos2, *ND3*_pos2, *ND6*_pos2, *ND2*_pos2 | GTR+I+G |
|  | P6: *CYTB*_pos1, *COX1*_pos1 | GTR+I+G |
|  | P7: *COX1*_pos2, *CYTB*_pos2 | GTR+I+G |
|  | P8: *COX1*_pos3, *COX2*_pos3, *CYTB*_pos3 | GTR+G |
|  | P9: *ND1*_pos1, *ND4*_pos1, *ND5*_pos1, *ND4L*_pos1 | GTR+I+G |
|  | P10: *ND1*_pos2, *ND4*_pos2, *ND5*_pos2, *ND4L*_pos2 | GTR+I+G |
|  | P11: *ND1*_pos3 | GTR+G |
|  | P12: *ND2*_pos3 | GTR+G |
|  | P13: *ND5*_pos3, *ND4*_pos3, *ND4L*_pos3 | HKY+G |
| **PCG12rRNA–ML** | P1: *ATP6*, *ATP8* | TIM+I+G |
|  | P2: *COX1* | GTR+I+G |
|  | P3: *COX3*, *COX2*, *CYTB* | GTR+I+G |
|  | P4: *ND4*, *ND1*, *ND5*, *ND4L* | GTR+I+G |
|  | P5: *ND2*, *ND3*, *ND6* | GTR+I+G |
|  | P6: *rrnL*, *rrnS* | GTR+I+G |
| **PCG12rRNA–BI** | P1: *ATP8*, *ATP6*, *ND6*, *ND3* | GTR+I+G |
|  | P2: *COX1* | GTR+I+G |
|  | P3: *COX3*, *CYTB*, *COX2* | GTR+I+G |
|  | P4: *ND1*, *ND4*, *ND5*, *ND4L* | GTR+I+G |
|  | P5: *ND2* | GTR+I+G |
|  | P6: *rrnL*, *rrnS* | GTR+I+G |
| **PCG12–ML** | P1: *ATP6* | TIM+I+G |
|  | P2: ATP8 | GTR+I+G |
|  | P3: *COX1* | GTR+I+G |
|  | P4: *COX3*, *COX2*, *CYTB* | GTR+I+G |
|  | P5: *ND1* | TVM+I+G |
|  | P6: *ND2*, *ND3*, *ND6* | GTR+I+G |
|  | P7: ND4, ND4L, ND5 | TVM+I+G |
| **PCG12–BI** | P1: *ND2*, *ATP6*, *ND6*, *ND3* | GTR+I+G |
|  | P2: *ATP8* | GTR+I+G |
|  | P3: *COX1* | GTR+I+G |
|  | P4: *COX3*, *COX2*, *CYTB* | GTR+I+G |
|  | P5: *ND1*, *ND4*, *ND5*, *ND4L* | GTR+I+G |
| **AA–ML** | P1: *ATP6*, *ATP8*, *COX2*, *COX3*, *CYTB* *ND2*, *ND3*, *ND6* | MTART+I+G+F |
|  | P2: *COX1* | MTART+I+G |

**Table S1.** *Cont.*

| **Data matrix** | **Subset Partitions** | **Models** |
| --- | --- | --- |
| **AA–ML** | P3: *ND1*, *ND4L*, *ND4*, *ND5* | MTART+I+G+F |
| **AA–BI** | P1: *ATP6*, *COX2*, *COX3* | MTREV+I+G |
|  | P2: *ATP8*, *ND2* | MTREV+I+G |
|  | P3: *COX1* | MTREV+I+G |
|  | P4: *ND1*, *CYTB* | MTREV+I+G |
|  | P5: *ND4L*, *ND6*, *ND3* | MTREV+I+G |
|  | P6: *ND5*, *ND4* | MTREV+I+G |
